# Deletion of Tfap2a in hepatocytes and macrophages promotes the progression of hepatocellular carcinoma by regulating SREBP1/FASN/ACC pathway and anti-inflammatory effect of IL10

**DOI:** 10.1038/s41419-025-07500-8

**Published:** 2025-04-03

**Authors:** Zhiwei Li, Chun Zhang, Guixiang Huang, Zixin Zhang, Qinghao Wang, Xiran Liu, Yanling Qin, Hao Zhou, Anyi Hou, Jun He, Limin Li, Xiang Hu, Xiaofeng Ding

**Affiliations:** 1 The National & Local Joint Engineering Laboratory of Animal Peptide Drug Development, College of Life Science, Hunan Normal University, Changsha, 410081 China; 2 Institute of Interdisciplinary Studies, Hunan Normal University, Changsha, 410081 China; 3 Hunan Provincial Key Laboratory of Regional Hereditary Birth Defects Prevention and Control, Changsha Hospital for Maternal & Child Health Care Affiliated to Hunan Normal University, Changsha, 410007 China; 4 College of Engineering and Design, Hunan Normal University, Changsha, 410081 China; 5 Peptide and small molecule drug R&D platform, Furong Laboratory, Hunan Normal University, Changsha, 410081 China

**Keywords:** Liver cancer, Tumour heterogeneity

## Abstract

The transcription factor AP-2α plays a crucial role in the control of tumor development and progression, and suppresses the proliferation and migration of hepatocellular carcinoma (HCC). However, the detailed function and mechanisms of AP-2α in the pathogenesis of HCC are still elusive. In the current study, we investigated the role of AP-2α regulation in liver injury-mediated HCC development. Downregulation of Tfap2a expression was found in the livers of DEN/CCl_4_-induced fibrosis and HCC mouse model. Hepatocyte (Alb-Cre), hepatic stellate cell (HSC) (Lrat-Cre) and macrophage (LysM-Cre) specific Tfap2a knockout mice were generated, respectively. Conditional knockout of Tfap2a was able to promote hepatic steatosis in Tfap2a^ΔHep^ and Tfap2a^ΔMΦ^ mice, but not in Tfap2a^ΔHSC^ mice fed with normal chow. Tfap2a^ΔHep^ and Tfap2a^ΔMΦ^ mice treated with DEN/CCl_4_ for 6 months increased tumor burden compared to Tfap2a flox controls. Tfap2a-deleted macrophages or hepatocytes could enhance lipid droplet (LD) accumulation in hepatocytes. Mechanistically, AP-2α binds to the promoter regions of SREBP1/ACC/FASN and inhibits hepatic lipid de novo synthesis. Deletion of Tfap2a in macrophages enhances polarization of M1 macrophages with increased iNOS expression but decreased CD206 expression, which resulted in increased pro-inflammatory cytokines and decreased anti-inflammatory factors, especially the hepatoprotective factor IL-10. The m6A modification writer WTAP could reduce the mRNA stability of AP-2α in a reader YTHDC1-dependent manner, whereas knockdown of WTAP or YTHDC1 enhances AP-2α expression and decreases lipid accumulation in HCC cells. Clinically, AP-2α expression negatively correlates with the expression of FASN, WTAP, YTHDC1 and the development of liver disease. Taken together, hepatocyte- or macrophage-specific deletion of Tfap2a promotes hepatic steatosis, fibrosis, and the development of HCC. These results suggest that AP-2α has been identified as a novel therapeutic target in fibrosis and inflammation-related HCC, exerting anti-lipogenesis, anti-inflammatory, and anti-tumor multi-roles.

## Introduction

Hepatocellular carcinoma (HCC) is the most prevalent primary liver malignancy and exerts a fatal cause of cancer-related deaths worldwide [1]. HCC is the fourth most common and second leading cause of cancer death worldwide, and mainly results from hepatitis B virus (HBV) infection as a major risk factor [2, 3]. Liver-specific metabolic alterations are distinctive features of the population with HBV-related HCC, such as increased glucose metabolism and overall activation of lipid biosynthesis [4]. Genes involved in fatty acid (FAs) biosynthesis are generally upregulated in most HCC tissues compared with noncancerous tissues [5]. Normal cells preferentially utilize circulating exogenous lipids, whereas cancer cells, including HCC cells, display high rates of de novo lipid (DNL) synthesis [6]. The synthesis of hepatic FA and triglyceride (TG) is mediated by SREBP1, SCD1, ACC1, fatty acid synthase (FASN) and PPARγ, which function in the regulation of FA and cholesterol biosynthesis and energy distribution [7–9]. The transcription factor SREBP1 mediates the downstream genes ACC and FASN in the pathway of FA chain modification or FA storage [10, 11]. ACC is the rate-limiting step of FA anabolism by converting acetyl-CoA to malonyl-CoA [8]. FASN as a crucial enzyme catalyzes the biosynthesis of fatty acids [12]. Currently, research on developing inhibitors against ACC and FASN to resist hepatic lipid accumulation has attracted much attention.

HCC development is not only originated from hepatocyte transformation but also closely associated with the interaction between hepatocytes and the hepatic immune microenvironment [13]. When hepatocytes, the main functional cells of the liver, are stimulated by fatty liver, they release pro-inflammatory cytokines to recruit the key immune cells such as hepatic macrophages (also known as Kupffer cells) into the tumor microenvironment [14]. Macrophages promote the proliferation and survival of hepatocytes by secreting cytokines including TNF-α and interleukin-6 (IL-6), which favorably supports the growth of HCC cells [15]. Moreover, hepatocytes could influence the polarization state of macrophages by releasing signaling molecules during the progression of HCC [16]. Macrophages usually exhibit M2-type features secreting IL-10, transforming growth factor β (TGF-β), and matrix metalloproteinase 2 (MMP-2) in HCC, which have immunosuppressive and tumor-promoting effects. M1 subtype macrophages participate in pro-inflammation, liver lipid accumulation, and tumor suppression by secreting IL-1β, IL-6, and TNF-α [17]. This M1/M2 shift promotes the invasion and metastasis of HCC, and inhibits the anti-tumor immune response [18]. Overall, hepatocytes and hepatic macrophages play a dual role in the occurrence of HCC, and the deep investigation of these processes is significant for the development of new therapeutic strategies targeting HCC.

Activator protein 2α (AP-2α) functions as a tumor regulator in solid cancers mainly by interacting with specific partners and binding promoter regions of critical downstream genes [19–21]. AP-2α regulates the hallmarks of tumors such as proliferation, invasion, stemness, and immune evasion [20, 22]. Our previous study has shown that AP-2α suppressed HCC cell proliferation and invasion [19]. However, the significance of AP-2α in inflammation-related HCC and lipid metabolism has not yet been investigated. The CCAAT/enhancer-binding protein (C/EBP) family controls SREBP1c expression during adipogenesis and lipogenesis [23]. C/EBPα mediated the terminal differentiation of adipocytes and AP-2α delayed the expression of C/EBPα and impaired the accumulation of TG [24]. AP-2α modulates the expression of lipid droplet proteins and induces lipid droplet accumulation in fibroblast cells in response to Wnt stimulation [25]. Taken together, these studies suggest that AP-2α is closely associated with TG synthesis and LD formation and growth.

Here, we investigated the importance of AP-2α in liver steatosis and inflammation by establishing Tfap2a-knockout mouse models in hepatocytes and macrophages. DEN/CCl_4_-induced Tfap2a^ΔHep^ and Tfap2a^ΔMΦ^ mice were found to enhance HCC tumorigenesis by improving DNL synthesis with the activation of SREBP1/ACC/FASN in steatotic hepatocytes and increasing the secretion of inflammatory cytokines in macrophages, respectively. AP-2α binds directly to the promoters of SREBP1/ACC/FASN, and suppresses their transcriptional activities in hepatocytes, but increases IL10 expression by directly binding to the promoter region of the IL10 gene. Finally, WTAP could degrade AP-2α mRNA in HCC by m6A RNA modification in a YTHDC1-dependent manner. These results demonstrated that the anti-steatosis and anti-inflammation of AP-2α in the liver, indicate the distinct roles and specific mechanisms of AP-2α during the progression of inflammation-related HCC.

## Methods

### Animal models

The loxP elements were inserted into the left side of the third exon and the right side of the fifth exon of Tfap2a using the CRISPR/Cas9 system (GemPharmatech, Jiangsu, China). Homozygous Tfap2a^fl/fl^ mice were crossed with Alb-Cre, Lrat-Cre, and LysM-Cre mice (GemPharmatech) to generate Tfap2a^ΔHep^, Tfap2a^ΔHSC^, and Tfap2a^ΔMΦ^ mice (all mouse backgrounds were C57BL/6JGpt), which were detected by genotyping. Only littermates with the flox allele served as corresponding wild-type controls (Tfap2a^fl/fl^). For the DEN/CCl_4_-induced HCC model, fourteen-day-old C57BL/6J mouse pups were injected intraperitoneally with DEN (50 mg/kg in normal saline) (N0756, Sigma-Aldrich) followed by CCl_4_ (10% CCl_4_, 0.5 μL/g in corn oil) (C14404678, Sigma-Aldrich) twice weekly for up to 24 weeks [26]. The same amount of corn oil or normal saline was injected into the control mice. The ethics committee from Hunan Normal University approved all mouse experiments.

### Isolation of primary mouse hepatocytes and macrophages

Primary hepatocytes were isolated from anesthetized 6-week-old mice. The hepatic portal vein was carefully punctured with a needle, the inferior vena cava was cut off, and the liver was flushed with the peristaltic pump as described [27]. The liver was then perfused with 3% collagenase IV (A004186, Sangon). The upper end of the liver was clamped and gently shaken to allow the hepatocytes to fall off the tissue. The cell suspension was filtered in a 100 μm-cell strainer (BS-100-XBS, Biosharp), centrifuged, suspended with 40% Percoll solution (P8370, Solarbio), and resuspended in DMEM complete medium (2319116, Vivacell) in a cell incubator at 37°C for 6 h.

Peritoneal macrophages were isolated from 6-week-old mice injected intraperitoneally with 3% Brewer thioglycolate medium for 3 days. Then, 8 mL of ice-cold DMEM containing 10% FBS (Gibco) was injected into the abdomen of sacrificed mice as described [28]. The exudates were centrifuged, resuspended in RPMI1640 medium (2320139, Vivacell) and seeded in culture dishes for 2 h. The remaining adherent cells are macrophages. Bone marrow cells were isolated from anesthetized 6-week-old mice. The mouse femur was taken and repeatedly rinsed with RPMI 1640 medium from the bone cavity. The washes were centrifuged and cultured for 3 days in medium containing 10 ng/mL M-CSF (M10014, Abmole). The adherents become mature macrophages on day 7.

### Cell culture and transfection

Hepatocellular carcinoma cell lines MHCC97H and Huh7 were purchased and authenticated from American Type Culture Collection (Rockville, MD). The cells were grown in incubators containing 5% CO_2_ at 37°C and were cultured in FBS-containing DMEM. Plasmids and siRNAs were transfected with Lipofectamine 3000 (L3000015, Invitrogen) following the standard protocol.

### Biochemical analyses

The livers of mice were homogenized and centrifuged. Protein concentration was determined using the Pierce BCA kit (Thermo Scientific), and AST (C010-2-1) and ALT (C009-1-1, Jiancheng, Nanjing) were detected following the manufacturer’s protocols. For the in vitro detection of lipid accumulation, primary hepatocytes or hepatocytes co-cultured with macrophages were stimulated with 0.5 mM palmitic acid (PA, A600497) and 1 mM oleic acid (OA, A502071, Sangon) in DMEM medium for 24 h, followed by Oil Red O staining (LJ0114B2010J, Bio Basic Inc). The cells or tissue sections were fixed and stained with Oil Red O for 20 min. Hematoxylin was added to stain the nuclei. Slides were mounted and observed with an Olympus microscope model CX41 (Tokyo, Japan). The levels of serum glucose, TG, free fatty acid (FFA) and total cholesterol (T-CH) (Jiancheng) of the livers were determined according to the experimental protocols.

### Luciferase assays, EMSA and ChIP

The transcription factor AP-2 binding sites were predicted in the promoter regions of SREBP1, FASN, ACC, IL-10, TNF-α and IFN-γ using the JASPAR website. m6A modification sites were predicted in the 3´ untranslated region (3´UTR) of AP-2α by the SRAMP website. These regions were amplified by PCR and inserted into the pGL3-basic vector. For the luciferase assays, HEK293 cells were transfected with the luciferase reporter plasmids and pCMV-Myc-AP-2α or WTAP/YTHDC1 siRNAs, and luciferase activities were measured using the luciferase reporter assays (E1500, Promega) as described [20]. The mutated binding sites (MUT) were designed based on the predicted AP-2 binding site (WT). After labeling the WT and MUT sequences (Supplemental Table 1) with biotin, EMSAs were performed with the EMSA Kit (GS008/9, Beyotime, Shanghai, China) as reported [22]. ChIP was performed using a ChIP assay kit from Beyotime (P2078) [22]. DNA-protein extracts were immunoprecipitated with rabbit polyclonal antibodies against AP-2α (YT0253, Immunoway), m6A (A19841, ABclonal), WTAP (A14695, ABclonal) or preimmune IgG (sc-66931, Santa Cruz Biotech, CA). The eluted DNA was amplified with ExTaq DNA polymerase (ABclonal). The PCR Primers were listed in Supplemental Table 1.

### qRT-PCR and Western blotting

For qRT-PCR, total RNA was extracted with TRIzol reagent (9109, TakaRa) and transcribed into cDNA using N6 random primers (Sangon) and GoScript™ Reverse Transcription System (Promega, A5000). Real-time PCR was performed using the SYBR green PCR Master Mix (DRR820A, TakaRa) with the ABI 7900 thermocycler (Thermo Fisher Scientific). For Western blotting, cells and tissues were lysed with the RIPA lysis (BL651A, Biosharp), lysates were denatured and loaded onto SDS-PAGE gels, membranes were transferred, blocked, and incubated with primary/secondary-antibodies, ECL chemiluminescence analysis (P10300, NCM) was performed to capture photographs. For detection of Apob48 in mouse liver tissues, the transfer buffer was modified (40 mM Tris, 20 mM sodium acetate, 2 mM EDTA, pH 7.4, 10% v/v methanol, 0.005% w/v SDS) and protein transfer was performed at 350 mA for 6 h at 4 °C [29]. Primary antibodies are AP-2α (3215, Cell Signaling Technology), SREBP1c (YT6055, Immunoway), ACC1 (A15606, ABclonal), FASN (A0461, ABclonal), ACLY (A22273, ABclonal), SCD1 (A26246, ABclonal), APOB48 (A4184, ABclonal), ACOX1 (A21217, ABclonal), Tubulin (AF7010, Affinity Biosciences), β-actin (P17001, ProMab Biotechnologies) and GAPDH (200306-7E4, ZEN-bio).

### Immunohistochemistry (IHC), Immunocytochemistry (ICC), Hematoxylin-eosin staining (H&E) and Transmission electron microscopy (TEM)

Human and mouse liver tissues were analyzed for IHC or ICC as previously described [22]. The primary antibodies used were AP-2α (3B5, Santa Cruz Biotech), WTAP, FASN, ACC, F4/80 (A18637, ABclonal), Desmin (A0699, ABclonal), CD206 (A8301, ABclonal), iNOS (340668, ZEN-bio) or IgG control (ab37355, Abcam). Liver tissues were analyzed by H&E and Sirius red staining according to the standard protocol. For TEM, approximately 1 mm^3^ of liver tissues were removed immediately after the mice were euthanized, and fixed in fixative at room temperature for 2 h, and then stored at 4 °C. The tissue samples were analyzed by TEM (Pinuofei Biotechnology Company, Wuhan, China) [30]. The ethics committee from Hunan Normal University approved the experiments, and all patients gave informed consent.

### FACS

Induced macrophages were detected by FACS analysis as described [31]. Primary antibodies are PerCP anti-mouse MHC II (E-AB-F0990F, Elabscience), APC anti-mouse CD206 (E-AB-F1135E, Elabscience), PE anti-mouse F4/80 (E-AB-F0995D, Elabscience) and FITC anti-mouse/human CD11b(E-AB-F1081C, Elabscience). The acquired data were processed using Flowjo software.

### Lipidomic analysis

Fresh liver tissues were placed in a centrifuge tube and then frozen at -80 °C. Non-targeted lipidomics analysis were performed by Majorbio Bio-Pharm Technology Co., Ltd (Shanghai, China) [32].

### RNA blots, methylated RNA immunoprecipitation (MeRIP), RIP

For RNA blots, total RNA was isolated to purify the mRNA according to the instructions of mRNA Isolation System (Z5210, Promega). The RNA secondary structure was destroyed at 95 °C for 3 min. mRNA was dropped onto the positively charged nylon membrane, which was then cross-linked and blocked. The membrane was then incubated with the m6A antibodies at 4 °C overnight and the secondary antibodies for 1 h at room temperature. The membrane was developed in the dark followed by chemiluminescence imaging and then stained with methylene blue to quantify the mRNAs. For MeRIP, the m6A antibodies and IgG were added to the fragmented mRNA sample and incubated at 4 °C for 2 h. Then the pierce Protein A/G magnetic beads (Thermo Fisher Scientific) were added and incubated at 4 °C for a further 2 h. After washing the beads, elution buffer was added at 4 °C as described [33]. The centrifuge tube was then placed on the magnetic stand for 1 min and the supernatant was retained, precipitated and dissolved in DEPC water. The RNA was transcribed into cDNA and AP-2α 3′UTR was amplified and detected by agarose gel electrophoresis. For RIP, the collected cells were suspended in 1× polymer lysis buffer on ice for 5 min. 100 μL of the supernatant was incubated with magnetic beads pre-incubated with IgG or WTAP, YTHDC1 antibodies (A7318, ABclonal) in 1×IP buffer overnight at 4 °C. The sample was then placed on a magnetic stand. The supernatant was discarded and the RIP sample was washed with NT-2 buffer as indicated [34]. The sample was digested with proteinase K buffer at 55 °C for 30 min. The supernatant was transferred and washed, the RNA was extracted and reverse-transcribed followed by PCR amplification.

### Statistical analysis

All statistical analyses were performed with GraphPad Prism 6 (GraphPad Software, San Diego, CA, USA). Data were expressed as mean ± SD of three independent experiments. Student’s t-test was used to compare two separate groups, and two-way ANOVA was performed to analyze more than two groups. For all tests, significance was set at P < 0.05 (*), < 0.01 (**), or < 0.001 (***).

## Results

### The expression of Tfap2a is downregulated in DEN/CCl4-induced HCC mouse models

**Fig. 1:**
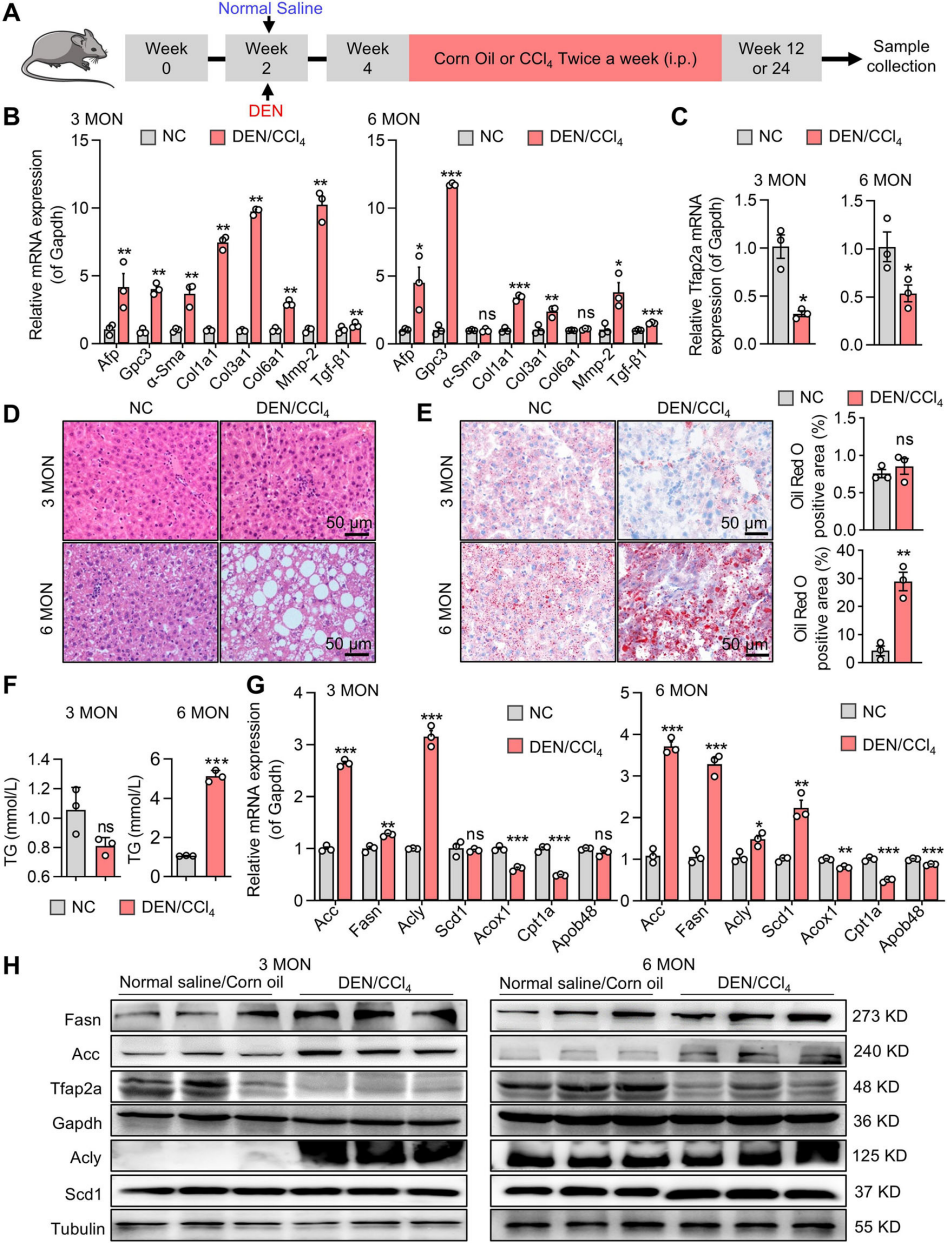
Downregulation of Tfap2a expression in DEN/CCl_4_-induced HCC models. **A** The strategy of DEN/CCl_4_-induced liver fibrosis at 3 months and HCC models at 6 months. **B** qRT-PCR analysis of target gene expression in the livers with fibrosis and HCC. **C** qRT-PCR analysis of Tfap2a expression in hepatic fibrosis tissues and HCC tumor tissues. **D**, **E** H&E and Oil Red O staining showing hepatocyte morphology and lipid droplet areas. **F** TG content in liver tissues from fibrosis and HCC models. **G**, **H** qRT-PCR and Western blot analysis of mRNA and protein levels. *, **, *** mean *P* < 0.05, *P* < 0.01, and *P* < 0.001. ns, no significance.

AP-2α has an inhibitory effect on the progression of HCC [19], but it is unclear that the specific role of AP-2α on the occurrence and development of primary liver cancer. We constructed a DEN**/**CCl_4_-induced fibrosis and inflammation-related primary HCC mouse model (Fig. 1A), which shows the progression of liver cancer as reported [35]. We found that AST and ALT levels were significantly increased in the livers of induced mice after 3 months and continued to increase after 6 months (Fig. S1A). Sirius staining showed that DEN/CCl_4_-induced livers exhibited collagen deposition (Fig. S1B), indicating liver fibrosis. Accordingly, we found increased mRNA levels of liver fibrosis (Col1a1, Col3a1, α-Sma, Tgf-β1) and HCC markers (Afp and Gpc3) at 3 months and 6 months (Fig. 1B), suggesting the successfully constructed DEN**/**CCl_4_-induced liver fibrosis and primary HCC model. We also found that the mRNA levels of transcription factor Tfap2a were lower than those in the control group (Fig. 1C). Pathological analysis showed that the positive area of lipid droplets was significantly increased in the H&E- and Oil Red O-stained livers of DEN**/**CCl_4_-injected mice at 6 months (Fig. 1D, E), and hepatic TG content was also increased (Fig. 1F), suggesting that induced primary HCC exhibited abnormal lipid metabolism in the livers. This construction was consistent with the pathological characteristics of previous studies of DEN/CCl_4_-induced mice [36], in which obvious lipid droplets were observed in H&E staining. The balance of lipid metabolism was maintained by lipid de novo synthesis, β-oxidation, lipid trafficking and lipid cleavage [37]. The mRNA expression of lipid-related genes was changed in the induced livers, especially the genes related to lipid de novo synthesis (Fig. 1G). The expression of lipid de novo synthesis-related proteins (Acc, Fasn and Acly) was increased in the induced livers at 3 months. Scd1, the rate-limiting enzyme for conversion of saturated fatty acids to monounsaturated fatty acids and a key protein for determination of TG accumulation [38], showed no significant expression changes between the induced and control liver. The expression of Acc, Fasn and Scd1 proteins in the liver of mice was significantly increased in the induced group compared with the control group at 6 months, while Acly, which converts citrate produced by the glycolysis-TCA cycle into acetyl-CoA, was slightly changed (Fig. 1H). The expression of Acox1 and Apob48 expressions was significantly decreased in the induced livers at 6 months, indicating impaired β-oxidation and lipid transport (Fig. S1C, D). Therefore, we hypothesized that the development of liver injury/fibrosis/HCC in the livers was associated with the downregulation of AP-2α expression and that there was a negative correlation between AP-2α and the DNL pathway.

### Deletion of Tfap2a in the hepatocytes and macrophages promotes hepatic steatosis

**Fig. 2:**
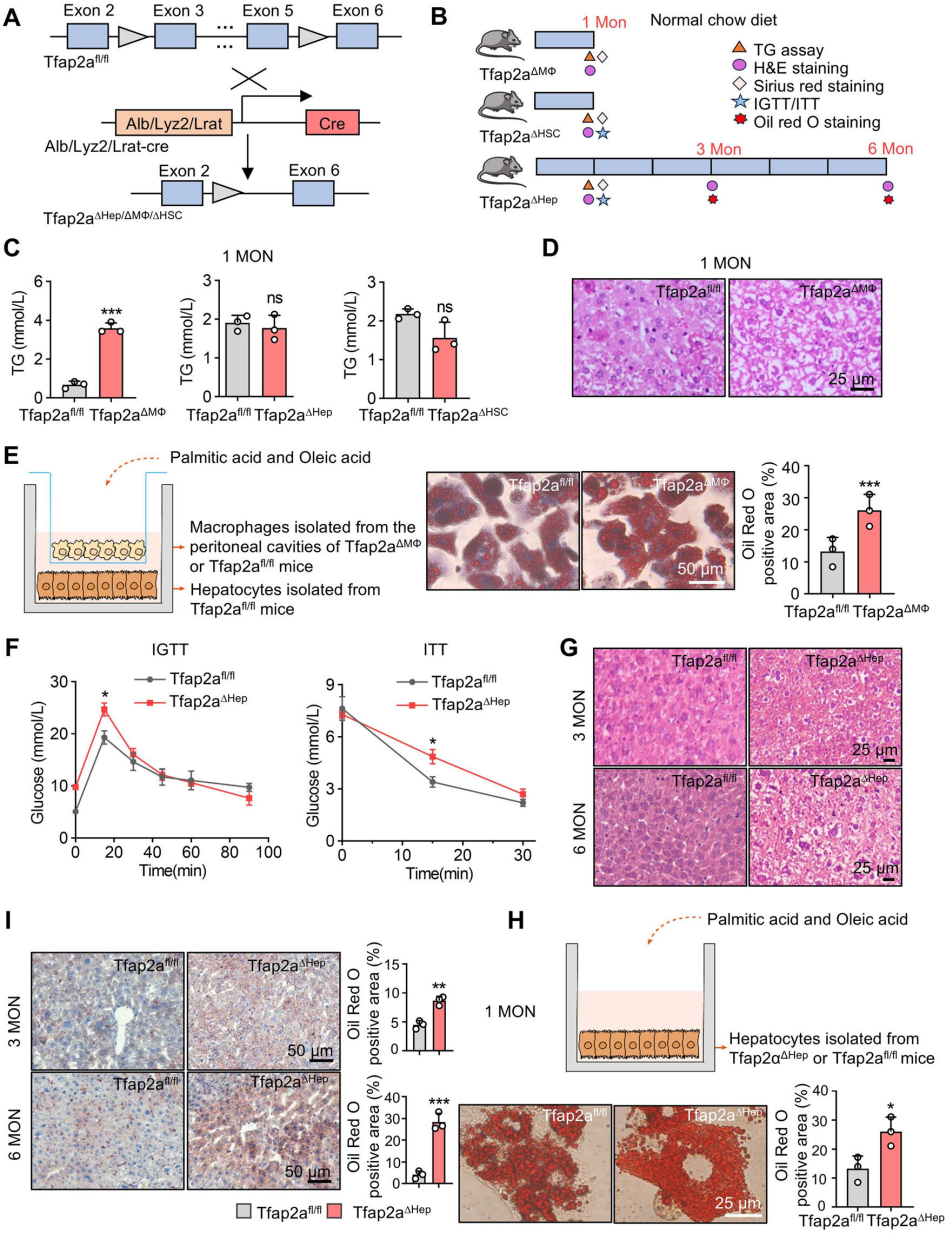
The effects of Tfap2a knockout on liver function. **A** The construction strategy of Tfap2a knockout mice in hepatocytes, macrophages and HSCs. **B** Strategies for the study of Tfap2a knockout mice under normal chow diet. **C** The TG contents in the livers of Tfap2a^KO^ mice. **D**, **E** H&E staining showing hepatocyte morphology and lipid droplet accumulation in Tfap2a^ΔMΦ^ mouse livers at 1 month or in cocultured hepatocytes. **F** IGTT and ITT analysis of glucose change in Tfap2a^ΔHep^ mice at 1 month. **G** H&E staining showing the morphological changes in the livers of Tfap2a^ΔHep^ mice at 3 and 6 months. **I**, **H**, Oil Red O staining of mouse livers at 3 and 6 months and of hepatocytes from WT or Tfap2a^ΔHep^ mice at 1 month. *, **, *** mean *P* < 0.05, *P* < 0.01, and *P* < 0.001. ns, no significance.

To explore the effects of AP-2α on the pathogenesis in primary HCC, we constructed Tfap2a knockout mice including parenchymal (hepatocytes) and non-parenchymal cells such as hepatic macrophages and hepatic stellate cells (Fig. 2A). The three knockout mice were subjected to the study strategy as shown in Fig. 2B. Tfap2a was successfully knocked out in three types of cells, and specific knockout mice were obtained (Fig. S2A–C). After one month of a normal chow diet, the body and liver weights of all mice showed no significant difference (Fig. S2D, E). There were unchanged ALT and AST levels in all mice, indicating that the livers of the Tfap2a^KO^ mice were not damaged (Fig. S2F, G). Sirius red staining showed no collagen deposition in Tfap2a specific knockout mice (Fig. S2H), suggesting that fibrosis did not occur. TG content only in the livers of Tfap2a^ΔMΦ^ mice was increased (Fig. 2C), and H&E staining showed that the livers of Tfap2a^ΔMΦ^ mice were accompanied by balloon-like swelling and LD abundance increase (Fig. 2D). The positive area of lipid droplets in the hepatocytes was increased in coculture with the Tfap2a-null intraperitoneal macrophages (Fig. 2E). However, there was no corresponding change in LDs in 1-month-old Tfap2a^ΔHep^ and Tfap2a^ΔHSC^ mice (Fig. S2I). Glucose and insulin tolerance tests showed that 1-month-old Tfap2a^ΔHep^ exhibited improved glucose tolerance and insulin resistance compared to wild-type mice, but not Tfap2a^ΔHSC^ mice (Figs. 2F and S2J). In addition, H&E and Oil red O staining showed that the livers of Tfap2a^ΔHep^ mice were accompanied by balloon-like swelling (Fig. 2G) and LD accumulation (Fig. 2I) at 3 and 6 months, which was considered a lesion of dysregulated liver lipid metabolism. Next, we isolated primary hepatocytes to determine whether deletion of Tfap2a leads to LD accumulation in the liver. Under PA and OA stimulation, the positive area of lipid droplets in Tfap2a knockout hepatocytes was increased (Fig. 2H). These results suggest that knockout of Tfap2a in macrophages and hepatocytes may lead to hepatocyte TG accumulation and impede hepatic lipid metabolism, whereas deletion of Tfap2a in HSCs has no significant effect on mouse livers.

### Loss of Tfap2a in the macrophages leads to M1 macrophage polarization and accelerates the occurrence of DEN/CCl_4_-mediated HCC

**Fig. 3:**
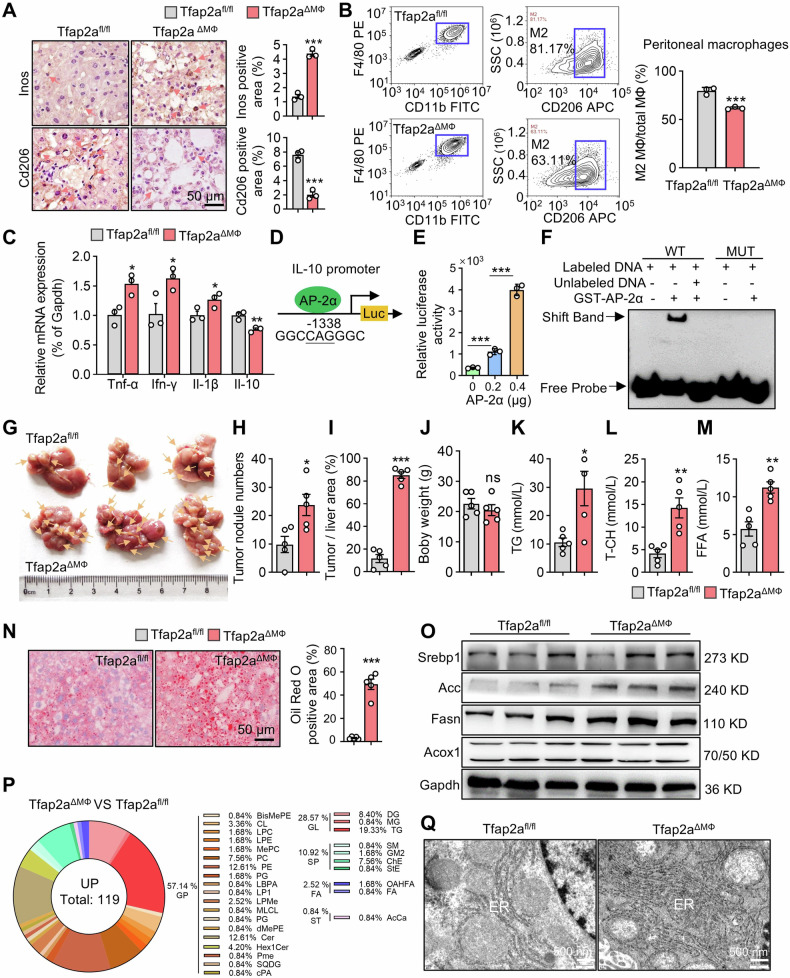
The effect of Tfap2a knockout in macrophages on DEN/CCl_4_-induced HCC models. **A** IHC analysis showing the effects of Tfap2a knockout in macrophages on the expression of macrophage marker genes. **B** FACS analysis detecting the effects of Tfap2a knockout in peritoneal macrophages on the proportion of M2 macrophages. **C** qRT-PCR analysis showing the expression of Tfap2a knockout in macrophages on the expression of pro-inflammatory and anti-inflammatory factors. **D** The predicted AP-2 binding site at the IL-10 promoter. **E** Luciferase assays demonstrating the effects of AP-2α on the transcriptional activities of IL-10 promoter. **F** EMSA showing the binding of AP-2α to IL-10 promoter in vitro. **G** The representative figures show the livers of Tfap2a^ΔMΦ^ mice and control mice. **H** Numbers of liver tumor nodules in Tfap2a^ΔMΦ^ mice (*n* = 5) and controls (*n* = 5). **I** Analysis of the tumor-to-liver area ratio based on liver morphology using Image J software. **J** Body weight of Tfap2a^ΔMΦ^ mice and control mice. **K**–**M** TG, T-CH and FFA levels in Tfap2a^ΔMΦ^ mice and control mice. **N** Oil Red O staining showing the positive area of lipid droplets from Tfap2a^ΔMΦ^ mouse livers. **O** Western blots of the effects of Tfap2a knockout in macrophages on DNL-related gene expression in livers. **P** Lipidomics analysis between Tfap2a^ΔMΦ^ and control mouse livers. **Q** TEM analysis of the structure of hepatocytes from Tfap2a^ΔMΦ^ and control mice. *, **, *** mean *P* < 0.05, *P* < 0.01, and *P* < 0.001. ns, no significance.

Deletion of Tfap-2a in macrophages leads to hepatic steatosis earlier than in hepatocytes. M1 macrophages promote the formation of lipid droplets in liver cells, leading to non-alcoholic steatohepatitis [16], which is accompanied by the secretion of proinflammatory cytokines [39]. We further explored the regulatory effect of AP-2α on macrophage polarization and the expression of proinflammatory cytokines secreted by macrophages. In Tfap2a^ΔMΦ^ mouse livers, IHC analysis revealed that the expression of M1 macrophage marker iNOS was increased while the M2 macrophage marker Cd206 was downregulated (Fig. 3A). Furthermore, we extracted peritoneal and bone marrow-derived macrophages from mice to induce macrophage polarization in vitro (Fig. S3A), and found that the percentage of M2 macrophages in Tfap2a knockout mice was lower than that of WT mice (Figs. 3B and S3B), demonstrating that Tfap2a knockout reduces the abundance of M2 macrophages. We found that Tfap2a knockdown in BMDMs significantly reduced mRNA levels of M2 polarization determinant PPAR-γ (Fig. S3C), while increased phosphorylated p65 levels (Fig. S3D), indicating NF-κB activation in inflammation. These data suggest that Tfap2a deficiency promotes M1-like macrophage polarization.

IL-10 has a hepatoprotective function against NASH and galactosamine/LPS-induced liver injury [40, 41]. IL-10 is considered to be an important linker that connects the anti-inflammatory function of Kupffer cells with the lipid metabolism of hepatocytes [42]. Inflammation-related cytokines were improved but anti-inflammatory cytokine IL-10 levels were reduced in the Tfap2a^ΔMΦ^ mouse livers by RT-qPCR analysis (Fig. 3C). We found that the promoter region of IL-10 contains a potential AP-2 binding site “GGCN_3_GGC” at position -1341 to -1333 relative to the translation initiation site (Fig. 3D). The luciferase assays showed that AP-2α enhanced the transcriptional activity of the IL-10 promoter (Fig. 3E), and the EMSA demonstrated that AP-2α proteins can directly bind to the IL-10 promoter region via wildtype AP-2-binding site, not the mutant AP-2 site (Fig. 3F). In addition, AP-2α proteins can directly bind TNF-α and IFN-γ promoter regions in vitro (Fig. S3F). These results indicated that AP-2α modulates inflammatory signaling pathways.

We constructed DEN**/**CCl_4_-induced Tfap2a^ΔMΦ^ mice and found that the induced Tfap2a^ΔMΦ^ mouse liver showed more tumor nodules (Fig. 3G, H), and the tumor-to-liver area ratio was increased (Fig. 3I), but DEN/CCl_4_-induced WT and Tfap2a knockout mice had no difference in body weight (Fig. 3J). TG,T-CH and FFA levels were improved (Fig. 3K–M) and the positive area of lipid droplets was increased (Fig. 3N) in DEN**/**CCl_4_-induced Tfap2a^ΔMΦ^ mouse livers. We speculated that hepatic TG accumulation was caused by the de novo lipid synthesis pathway. We found that the expression of Srebp1, Acc and Fasn was significantly upregulated in the livers of Tfap2a^ΔMΦ^ mice as compared to control mice, while Acox1 expression, a protein related to lipid oxidation, did not change (Fig. 3O). We performed the non-targeted lipidomic analysis of livers and found a total of 163 different lipids between Tfap2a^fl/fl^ and Tfap2a^ΔMΦ^ mice (Fig. S3G), of which 119 lipids were significantly up-regulated in Tfap2a^ΔMΦ^ mice. Classifying the up-regulated lipids, it was found that glycerolipids (GL), the products of de novo lipid synthesis, accounted for 28.57%, of which TG accounted for 19.33% of all up-regulated lipids (Fig. 3P). These results illustrate that knockout of macrophage AP-2a leads to massive accumulation of TG in mouse livers. Excessive lipogenesis can lead to endoplasmic reticulum stress [43] and TEM analysis showed the swelling and disorganized endoplasmic reticulum in the Tfap2a^ΔMΦ^ mouse livers (Fig. 3Q). Therefore, the deletion of AP-2α in macrophages drives the progression from inflammation and steatosis to HCC.

### Deletion of Tfap2a in hepatocytes accelerates the occurrence of DEN/CCl_4_-mediated HCC

**Fig. 4:**
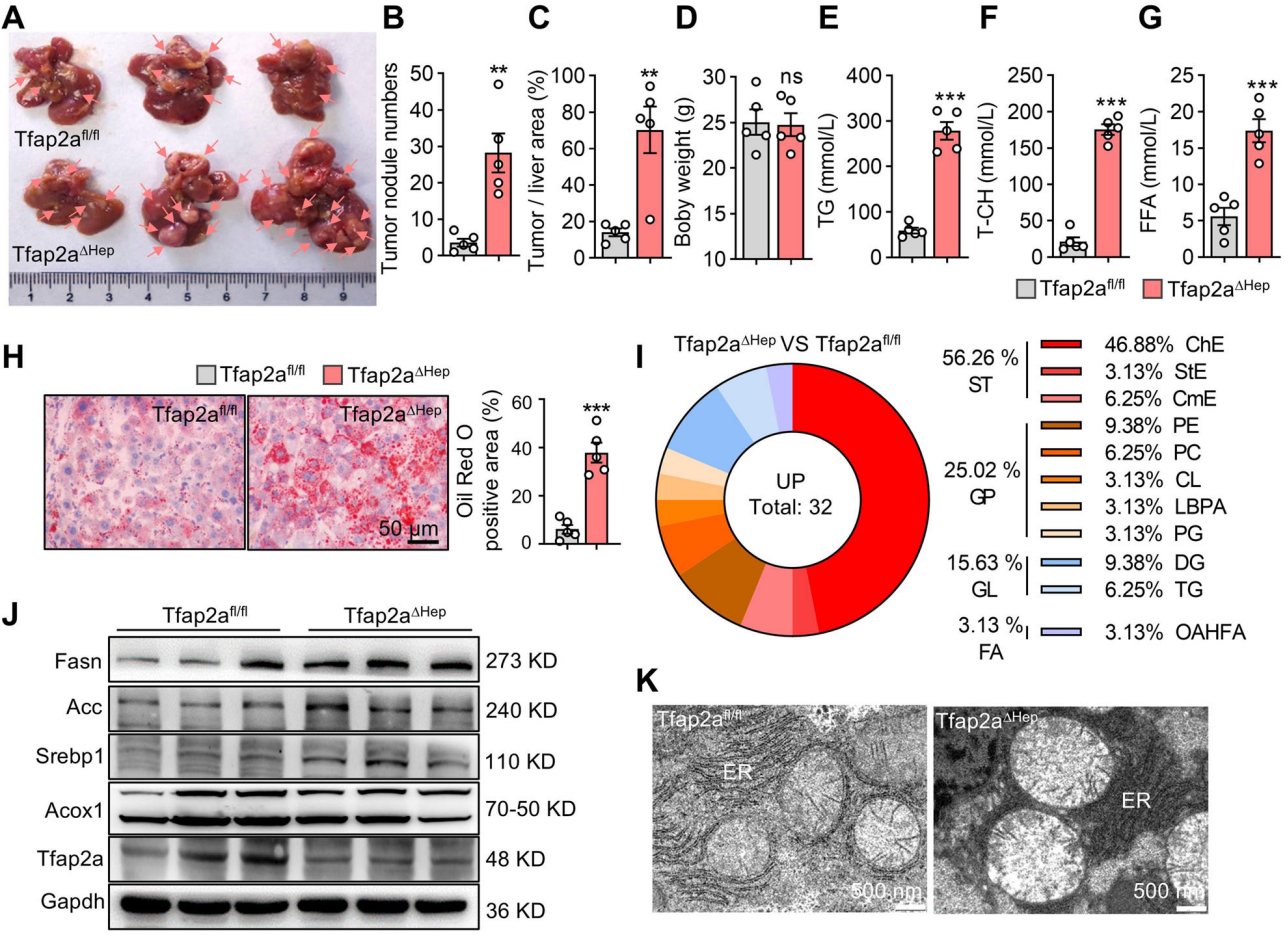
The effect of Tfap2a knockout in hepatocytes on DEN/CCl_4_-induced HCC models. **A** The representative images of the livers from Tfap2a^ΔHep^ mice and controls. **B**, **C** Numbers of liver tumor nodules and the tumor-to-liver area ratio in Tfap2a^ΔHep^ mice (*n* = 5) and control mice (*n* = 5). **D** Body weights in induced Tfap2a^ΔHep^ mice and controls. **E**–**G** TG, T-CH, and FFA levels in Tfap2a^ΔHep^ mice and control mice. **H** Oil Red O staining showing the positive area of lipid droplets in Tfap2a^ΔHep^ mice and control mice. **I** Lipidomics analysis of livers from Tfap2a^ΔHep^ and control mice. **J** Western blots of the effects of Tfap2a knockout in hepatocytes on DNL-related gene expression. **K** TEM analysis of the structure of hepatocytes from Tfap2a^ΔHep^ and control mice. **, *** mean *P* < 0.01 and *P* < 0.001. ns, no significance.

The above results have shown that a deficiency of Tfap2a in hepatocytes leads to hepatic steatosis. We wondered whether the deletion of Tfap2a in hepatocytes promotes primary liver cancer by affecting metabolism. We constructed a DEN**/**CCl_4_-mediated HCC model and found that the nodule number and the tumor-to-liver area ratio were increased in induced Tfap2a^ΔHep^ mice (Fig. 4A–C). There was no difference in body weight between Tfap2a^ΔHep^ and Tfap2a^fl/fl^ mice (Fig. 4D). However, we also found that the levels of TG, T-CH and FFA were correspondingly increased in the livers of induced Tfap2a^ΔHep^ mice (Fig. 4E–G). The number and positive area of lipid droplets were higher in the livers of induced Tfap2a^ΔHep^ mice than in those of Tfap2a^fl/fl^ mice (Fig. 4H). Non-targeted lipidomic analysis of Tfap2a^fl/fl^ and Tfap2a^ΔHep^ mouse livers yielded a total of 38 differential lipids (Fig. S4B). There were 32 significantly up-regulated lipids in the liver of Tfap2a^ΔHep^ mice. Sterol lipids (ST) accounted for 56.26% of all up-regulated lipids, of which cholesteryl ester (ChE) was the most abundant (46.88%), which is thought to be indirectly regulated by SREBP1c [44]. The proportion of glycerolipids (including DG and TG) was 15.63%, indicating that the de novo lipid synthesis pathway was enhanced in Tfap2a knockout mice (Fig. 4I). Deletion of Tfap2a in hepatocytes could activate the expression of proteins related to lipid de novo lipogenesis (Fig. 4J). As reported [45], TEM analysis showed that FFA-mediated lipotoxicity induces endoplasmic reticulum stress in Tfap2a^ΔHep^ mouse livers (Fig. 4K). These results indicated that under the induction of DEN/CCl_4_, the loss of Tfap2a in hepatocytes promotes the de novo lipid synthesis pathway, leads to hepatocyte TG accumulation, triggers fatty degeneration, and accelerates the formation of HCC.

### AP-2α binds to the promoters of the SREBP1/ACC/FASN genes and inhibits de novo lipogenesis

**Fig. 5:**
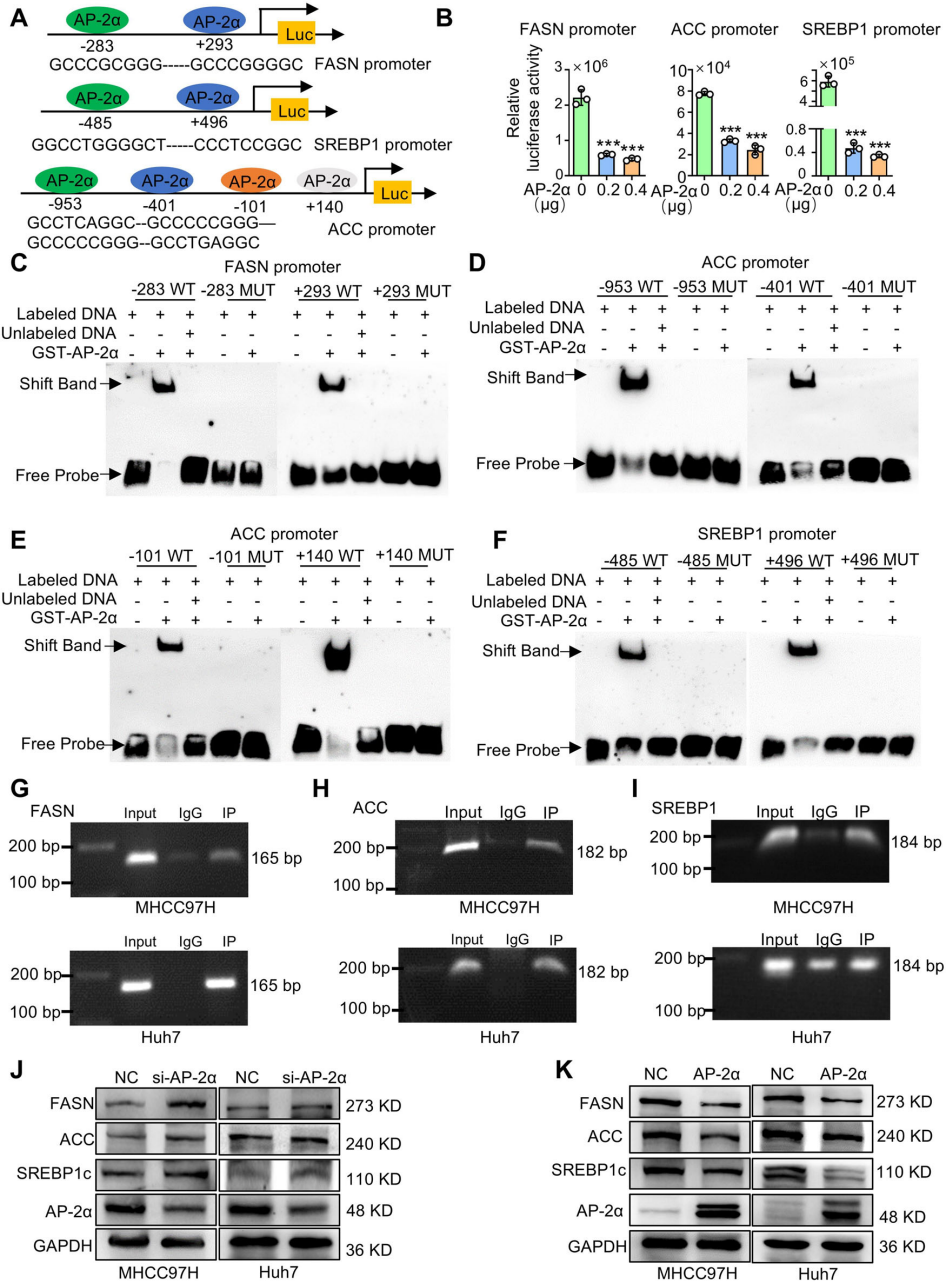
The regulatory effects of AP-2α on the promoters of FASN, SREBP1 and ACC. **A** The predicted AP-2 binding sites at the FASN, SREBP1 and ACC promoters. **B** Luciferase assays demonstrating the effects of AP-2α on the transcriptional activities of three promoters. **C**–**F** EMSA showing the binding of AP-2α to three promoters involved in DNL synthesis in vitro. **G**–**I** ChIP analysis demonstrating the binding of AP-2α to these promoters in MHCC97H and Huh7 cells. **J**, **K** Western blots demonstrating the effects of AP-2α knockdown/overexpression on DNL-related gene expression in MHCC97H and Huh7 cells. *** mean *P* < 0.001.

SREBP1, a critical transcriptional regulator in the DNL pathway, directly regulates the expression of ACC and FASN [44]. As a transcription factor, AP-2α proteins bind to the palindromic recognition sequence 5′-GCCN_3/4_GGC-3′ that has regulated transcriptional levels of target genes [46]. Next, we analyzed the promoter of SREBP1, FASN and ACC using the JASPAR website, and found the potential AP-2 binding sites (Fig. 5A). The pGL3-basic plasmids with SREBP1, FASN and ACC promoters were constructed, respectively. The reporter activities of promoter plasmids were reduced by AP-2α in a dose-dependent manner compared with the control group (Fig. 5B), suggesting that AP-2α can bind to the promoters of these genes, and subsequently inhibit their transcriptional activities. The binding ability of AP-2α to these sites was simulated by EMSA in vitro, and the results confirmed that the AP-2α protein binds to the candidate sites of these gene promoters, but not to their corresponding mutant sites (MUT) (Fig. 5C–F). Further ChIP analysis was performed to verify whether the AP-2α proteins bind to their promoters in HCC cells. The PCR results showed that these promoter DNA fragments could be immunoprecipitated with anti-AP-2α antibodies in HCC cells (Fig. 5G–I). Knockdown of AP-2α resulted in the upregulation of these proteins (Fig. 5J), while overexpression of AP-2α downregulated the protein levels of SREBP1c, ACC and FASN in HCC cells (Fig. 5K). Thus, AP-2α directly binds to the promoters of key genes in the DNL pathway and inhibits their transcriptional activities, thereby blocking the DNL pathway.

### m6A modification reduces the stability of AP-2α mRNAs in HCC

**Fig. 6:**
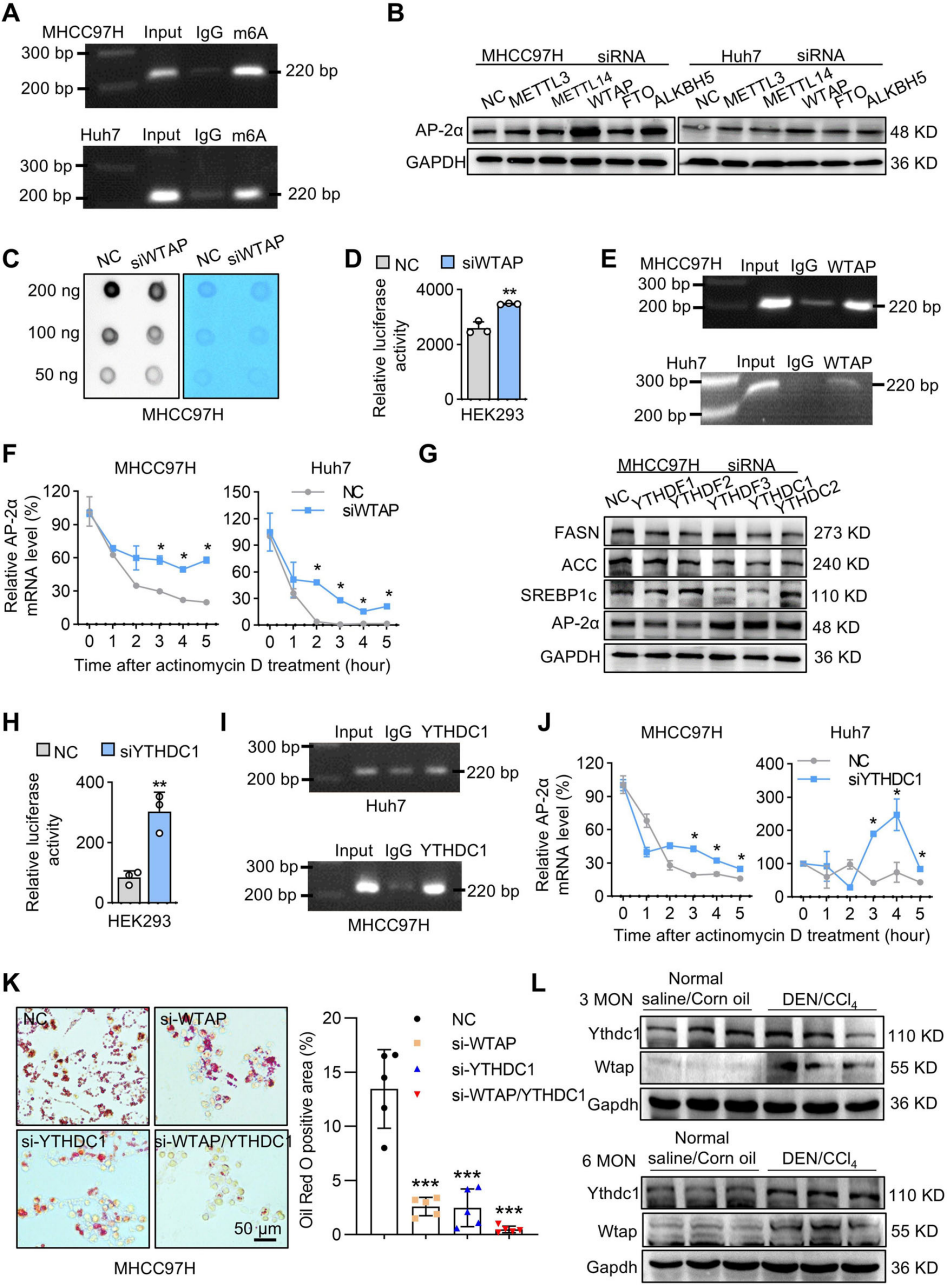
The effects of m6A RNA modification on AP-2α expression. **A** MeRIP assays showing that the m6A antibodies immunoprecipitate AP-2α 3′UTR with m6A binding sites in HCC cells. **B** Western blotting revealing the effects of knockdown of m6A regulators on AP-2α expression. **C** Dot blot analysis detecting the effect of WTAP siRNAs on the level of m6A modification. **D** Luciferase assays demonstrating the effect of WTAP siRNAs on AP-2α 3′UTR reporter activity. **E** RIP assays confirming the binding between WTAP antibodies and AP-2α 3′UTR. **F** qRT-PCR detecting the effect of WTAP knockdown on the half-life of AP-2α mRNAs. **G** Western blotting showing the effect of knockdown of m6A readers on the expression of AP-2α and DNL-related genes. **H** The effects of YTHDC1 siRNAs on AP-2α 3′UTR reporter activity. **I** RIP assays showing the binding between anti-YTHDC1 antibodies and AP-2α m6A sites. **J** The effect of YTHDC1 knockdown on the half-life of AP-2α mRNAs. **K** Oil Red O staining showing the effects of WTAP and YTHDC1 knockdown on lipid accumulation in MHCC97H cells. **L** Western blotting detecting Wtap and Ythdc1 expression in mouse livers from fibrosis and HCC models. *, ** mean *P* < 0.05 and *P* < 0.01.

Studies have shown that m6A can affect the occurrence and development of tumors including HCC [47]. TFAP2c, the transcription factor AP-2 family member, was methylated by METTL3 and recognized by IGF2BP1, which enhances the mRNA stability of TFAP2c, thereby making seminoma resistant to cisplatin [48]. We therefore investigated whether the dysregulation of Tfap2a expression was related to epigenetic modification. The m6A modification usually occurs in the coding sequence (CDS) and 3’UTRs of target genes [49, 50]. We found that the m6A sites on AP-2α CDS and 3’UTRs were located at +1947, +2062 and +2139 bp relative to the start site (ATG), respectively (Fig. S5A, B). MeRIP-qPCR experiments found that the m6A sites in 3’UTR of AP-2α gene were significantly enriched as compared with the control group in MHCC97H and Huh7 cells (Fig. 6A), indicating that m6A modification is involved in the regulation of AP-2α expression. Next, m6A methylase and demethylase were interfered with siRNAs to determine their effects on AP-2α protein levels. When WTAP was knocked down, AP-2α expression was upregulated in MHCC97H and Huh7 cells (Fig. 6B). m6A dot blots showed that WTAP siRNAs decreased the level of m6A modification in MHCC97H cells (Fig. 6C). Moreover, luciferase assays confirmed that WTAP siRNAs increased the reporter activity of the pGL3-AP-2α 3’UTR plasmids (Fig. 6D), suggesting that WTAP affects the expression of AP-2α through m6A modification. RIP-qPCR analysis revealed that the m6A sites in the 3’UTRs of AP-2α were effectively enriched by anti-WTAP antibodies as compared with the IgG group (Fig. 6E). To determine the effect of WTAP on the stability of AP-2α mRNAs, HCC cells were treated with actinomycin D under WTAP-interfering conditions. The WTAP siRNA group stabilized the AP-2α mRNAs and prolonged their half-life in both HCC cell lines (Fig. 6F). m6A modification must be mediated by “reader” proteins such as YTHDFs and IGF2BPs [51, 52]. Next, Western blots showed that AP-2α expression was upregulated and the SREBP1c/FASN/ACC pathway was inhibited only when YTHDC1 expression was knocked down in the MHCC97H cell lines (Fig. 6G). YTHDC1 interference increased the activities of AP-2α 3’UTR reporters (Fig. 6H). And anti-YTHDC1 antibodies could enrich m6A sites of AP-2α 3’UTRs (Fig. 6I). YTHDC1 siRNAs were able to reduce the decay of AP-2α mRNAs (Fig. 6J).

Next, we knocked down WTAP and YTHDC1 by siRNAs in MHCC97H cells and stimulated cells with PA and OA for 24 h. Oil Red O staining revealed that a single knockdown of WTAP or YTHDC1 could inhibit lipid droplet accumulation, and a double knockdown of WTAP and YTHDC1 more significantly inhibited the generation of lipid droplets (Fig. 6K). Consistently, immunoblotting results confirmed that compared to the NC group Wtap and Ythdc1 protein levels were increased in DEN/CCl_4_-induced mouse livers, especially at 6 months (Fig. 6L). This finding verified that WTAP suppressed the expression of AP-2α in an m6A-YTHDC1-mediated manner in the steatosis and inflammation-related HCC model.

### In clinical liver disease samples downregulation of AP-2α is closely associated with the upregulation of WTAP, YTHDC1 and FASN expression

**Fig. 7:**
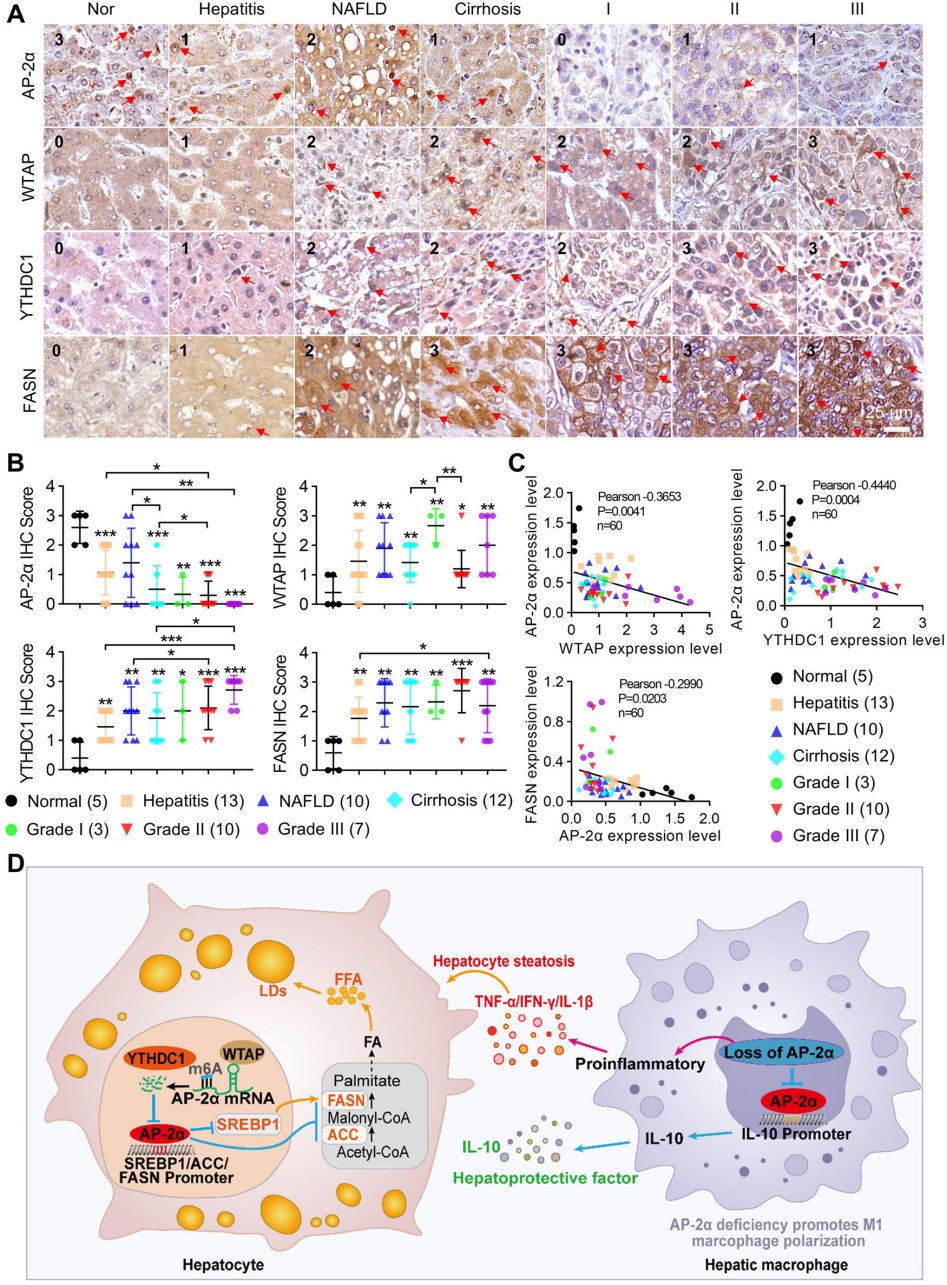
IHC analysis of the expression of AP-2α and its upstream/downstream genes at different stages of human liver diseases. **A** The expression of AP-2α, WTAP, FASN, and YTHDC1 in diseased liver tissues detected by IHC analysis. **B** The staining scores of the expression of AP-2α, WTAP, FASN, and YTHDC1 in normal and diseased liver tissues. **C** The expression correlation between AP-2α and upstream/downstream genes in diseased liver tissues. **D** The model figure shows the function and the regulatory mechanisms of AP-2α in liver diseases. Deletion of Tfap2a in hepatocytes and macrophages leads to inflammation, steatosis and progression from liver diseases to HCC. Mechanistically, AP-2α binds directly to the promoter regions of SREBP1/FASN/ACC, inhibits their transcription and subsequently suppresses hepatic DNL synthesis, thereby preventing lipid accumulation, and attenuating lipotoxicity-mediated ER stress. Deletion of Tfap2a in macrophages could increase iNOS expression but decrease Cd206 expression, which enhanced the polarization of pro-inflammatory M1 macrophages by decreasing the secretion of anti-inflammatory mediator IL-10. WTAP was able to reduce the stability of AP-2α mRNAs by m6A RNA modification in a YTHDC1-dependent manner. *, **, *** mean *P* < 0.05, *P* < 0.01, and *P* < 0.001.

We analyzed liver disease samples (Supplementary Tables 2–5) to confirm the function of AP-2α regulating liver lipid metabolism. AP-2α protein levels were gradually downregulated in NAFLD/NASH and HCC as compared to adjacent healthy liver tissues. However, the expression of WTAP, YTHDC1 and FASN was increased in NAFLD/NASH/HCC samples (Fig. 7A, B), indicating m6A-dependent downregulation of AP-2α during the inflammation-cancer progression. The expression of AP-2α correlated negatively with the expression of WTAP, YTHDC1 and FASN in liver disease samples (Fig. 7C). These data suggest that m6A RNA modification and AP-2α regulation play an important role in HCC progression.

## Discussion

Fatty liver diseases have been increasingly reported as one of the main risk factors for HCC, and liver inflammation, fibrosis and cirrhosis occur frequently during HCC development [2]. The fatty liver releases pro-inflammatory cytokines and causes inflammation with hepatocyte damage and regeneration, leading to cellular mutations that may eventually progress to HCC [53]. The molecular mechanisms of fatty liver and HCC, such as metabolic abnormalities, HSC activation, and oxidative stress, need to be urgently researched to find new targets for HCC intervention. In this study, we systematically investigated the specific function of Tfap2a in fibrosis and inflammation- or lipid metabolism disorder-related HCC by conditionally knocking out Tfap2a in three types of cells from mouse liver combined with DEN/CCl_4_ induction. Our data confirmed that deletion of AP-2α in hepatocytes and macrophages leads to disturbances in hepatic lipid metabolism, which are caused by different mechanisms of action. We detected the downregulation of Tfap2a expression in the mouse livers during the progression of chemically induced HCC. There is also a strong correlation between HCC progression and AP-2α downregulation in clinical liver disease samples. These findings indicate the downregulation of Tfap2a may be closely related to the progression of HCC associated with lipid metabolism disorders.

Under normal diet conditions, the body weight of 1-month-old Tfap2a knockout mice of all species did not change significantly compared with WT mice. Interestingly, Tfap2a^ΔMΦ^ mice had significantly increased TG levels and liver steatosis as compared with WT mice, suggesting that AP-2a loss in macrophages leads to lipid metabolism disorder. Studies have shown that M1 macrophages cause hepatocyte lipid metabolism disorders by secreting pro-inflammatory factors [54]. We found that Tfap2a-deficient macrophages exhibit decreased expression of the M2 marker Cd206 and increased expression of the M1 marker iNOS. We also demonstrated that loss of Tfap2a in macrophages inhibits the polarization of myeloid macrophages toward the M2 phenotype in vitro. Deletion of Tfap2a in macrophages enhanced NF-κB activity and suppressed PPARγ activation, which regulates the secretion of inflammatory cytokines produced by M1 macrophages and inhibits the phenotype of M2 macrophages [55]. We detected that the mRNA levels of pro-inflammatory factors (Tnf-α, Ifn-γ, Il-1β) were increased, while Il-10 mRNA was decreased in the liver of Tfap2a^ΔMΦ^ mice. IL-10 is an anti-inflammatory factor secreted by M2 macrophages and plays a protective role in the liver [56, 57]. Subsequently, luciferase assay and EMSA demonstrated that AP-2α positively regulates IL-10 transcription. We also found that the promoters of TNF-α and IFN-γ contain AP-2 binding sites, and verified their binding to AP-2α in vitro. These results indicate that macrophage AP-2α regulates the transcription levels of inflammation-related genes. Under DEN/CCl_4_ stimulation, more tumor nodules appeared in the liver of Tfap2a^ΔMΦ^ mice, and the levels of TG, T-CH, and FFA in the liver increased. Lipidomics also showed that the proportion of TG in the liver of Tfap2a^ΔMΦ^ mice increased significantly compared with that in Tfap2a^fl/fl^ mice. In addition, TEM results showed the phenomenon of endoplasmic reticulum stress in the liver of Tfap2a^ΔMΦ^ mice. Therefore, we speculate that the M1-like macrophages of Tfap2a^ΔMΦ^ mouse are continuously replenished by chemical toxins. M1 like macrophages secrete pro-inflammatory factors to stimulate hepatocytes, leading to disorders in hepatocyte lipid metabolism and thus triggering carcinogenesis.

1-month-old Tfap2a^ΔHep^ mice showed tolerance to glucose and resistance to insulin, and 3- and 6-month-old mice developed hepatic steatosis and lipid accumulation in the liver. Interestingly, the body weight of Tfap2a^ΔHep^ mice did not differ significantly at different ages as compared with WT mice. This suggests that the deletion of Tfap2a in hepatocytes does not lead to obesity, but affects the lipid metabolism pathway of the liver. The lipid metabolism disorder in the liver of Tfap2a^ΔHep^ mice is time-dependent, which is different from the Tfap2a^ΔMΦ^ mice that quickly show symptoms of liver steatosis at 1 month of age. For this phenomenon, we reasonably speculate that the loss of Tfap2a in hepatocytes will not quickly lead to chronic liver inflammation. Similarly, under DEN/CCl_4_ stimulation, the tumor nodules formed in the liver of Tfap2a^ΔHep^ mice, and the levels of liver TG, T-CH and FFA were significantly increased compared with Tfap2a^fl/fl^ mice. Unlike the lipidomics of the liver of Tfap2a^ΔMΦ^ mice, the liver of Tfap2a^ΔHep^ mice showed more steroid lipids, of which ChE accounted for 46.88% of all upregulated lipids. The proportion of DNL products DG and TG was also upregulated compared with Tfap2a^fl/fl^ mice. This result suggests that AP-2α may directly negatively regulate the SREBP1 and DNL pathways. Mechanisms of AP-2α-mediated lipogenesis showed that enhanced cholesterol and fatty acid synthesis in Tfap2a-deficient hepatocytes was associated with the upregulation of the SREBP1c/ACC/FASN axis, key regulators of hepatic de novo lipogenesis [58]. SREBP1/2 has been shown important regulators of cholesterol and fatty acid synthesis. FASN was considered as a potential target to block lipogenesis, including cancers. FASN expression in hepatocytes is upregulated in NAFLD patients [59, 60]. Some studies have reported that the recruitment of an E3 ligase regulates the degradation of FASN in NAFLD [61] and FASN inhibitors have shown therapeutic potential for NAFLD in clinical trials [62, 63]. However, hepatocyte-specific Fasn-knockout mice develop hypoglycemia and fatty liver [64]. Based on the heterogeneity of physiological and pathological properties of FASN, it is more emergent to identify new FASN regulators to combat liver disease-related metabolism. We found that AP-2α can directly bind to the promoters of SREBP1/ACC/FASN genes and inhibit their transcription and protein levels in HCC cells, leading to a reduction in hepatic fatty acid synthesis and excessive accumulation of lipid droplets (Fig. 7D), suggesting that de novo FA synthesis in steatotic hepatocytes is directly mediated by AP-2α. Increased fatty acid and sterol synthesis have been described in various cancers, indicating a carcinogenic role [65]. The expression of FASN is highly upregulated in malignant tumors and provides energy for tumor cell proliferation [12]. The pro-oncogenic enzyme FASN is involved in the initiation and metastasis of HCC [66, 67]. Progression of lipid metabolism disorder-related HCC is associated with the downregulation of AP-2α and the upregulation of SREBP1c/ACC/FASN in mice and patients. The active nuclear form of SREBP1 enhances ER stress and autophagy [68], while ER stress also in turn triggers SREBP1/2 to drive lipogenesis and steatohepatitis [69]. Consistent with this, swell and disorganization of ER were induced upon the deletion of Tfap2a in hepatocytes. Therefore, the link between ER stress and the regulatory network of AP-2α in the lipid metabolism disorder-related HCC process deserves further investigation.

M6A-related regulators play an important role in the pathogenesis and progression of HCC [70], exerting their potential diagnostic, prognostic and therapeutic value in HCC. m6A “writer” WTAP directed m6A modification and enhanced the oncogenesis of HCC, while silencing WTAP inhibited the proliferation and aggressiveness of HCC [71, 72], suggesting a prognostic biomarker and therapeutic target for HCC. We found that the knockdown of WTAP increases the stability of AP-2α mRNA by reducing m6A methylation in the 3′UTR of AP-2α, and enhances the tumor suppressive role of AP-2α. YTHDC1 is a member of the YTH domain-containing protein family and the only nuclear protein in this protein family that is involved in transcription, mRNA splicing and nuclear export [73–75]. Previous reports have shown that YTHDC1 shuttles between the nucleus and cytoplasm, and that cytoplasmic YTHDC1 protein may be involved in the processing of mature mRNAs [76]. For mature mRNA, YTHDC1 plays a regulatory role by controlling its stability. YTHDC1 recognizes METTL16-mediated 3’ UTR N6-methylation modification sites of MAT2A mRNA and promotes MAT2A mRNA degradation [77]. YTHDC1 promotes AKT phosphorylation by degrading PTEN mRNA, thereby alleviating ischemic stroke [78]. Moreover, YTHDC1 could destabilize a subset of m6A-marked chromatin-associated RNAs (caRNAs) (mainly LINE1 repeats) via the nuclear exosome targeting (NEXT) complex [73]. In this study, we demonstrated that YTHDC1 knockdown upregulates AP-2α protein expression and explained that the reason was that YTHDC1 regulated AP-2α mRNA stability as an m6A reader. However, it remains to be clarified whether the m6A-dependent degradation of AP-2α mRNA is due to the binding of nuclear YTHDC1, leading to the maintenance of heterochromatin at AP-2α locus. Interestingly, recent studies suggest that YTHDC1 enhances the stability of the lncRNA LINC00294 after METTL3-mediated m6A modification, thereby promoting the glycolysis pathway and growth of HCC cells [79]. We found that YTHDC1 knockdown also inhibited lipid droplet accumulation in liver cancer cells, indicating that YTHDC1 was a key protein in lipid metabolism by participating in RNA modification. Metabolic dysregulation drives the development of HCC from tumor initiation to progression, and targeting abnormal metabolism indicates new strategies for HCC therapy [80]. Therapeutic administration of targeting WTAP might suppress HCC progression in DEN/CCl_4_-fed mice, and the WTAP-targeted AP-2α axis becomes a novel option for the treatment of lipid metabolism disorder-induced fibrosis and HCC. During the specific process from NAFLD to HCC, the AP-2α-mediated specific molecular network needs further delineated in the future.

In conclusion, our work provides evidence that a novel regulator AP-2α in lipid metabolism disorder-related HCC exerts a suppressive role in DNL synthesis and the transition from inflammation to cancer in HCC, with potential implications for the treatment of hepatic steatosis and HCC. AP-2α functions as a critical suppressor of SREBP1c/ACC/FASN-mediated lipogenesis in hepatocytes, which is attenuated by the m6A “writer” WTAP, and as that of pro-inflammatory M1 macrophages by activating the expression of anti-inflammatory cytokine IL10. Clarifying the specific role and intricate mechanisms of AP-2α will shed light on the therapy of steatosis-related liver diseases.

## Supplementary information

Supporting Information

Original data of WB

## Data Availability

Data generated or analyzed during this study are available from the corresponding author upon reasonable request.
