# Supplementary material for: Deletion of Tfap2a in hepatocytes and macrophages promotes the progression of hepatocellular carcinoma by regulating SREBP1/FASN/ACC pathway and anti-inflammatory effect of IL10
Source: Cell Death Dis. 2025 Apr 3;16(1):245. doi: 10.1038/s41419-025-07500-8 (PMC11968862; doi:10.1038/s41419-025-07500-8)
Supplement: Supplementary file 1 — Supporting Information [file 41419_2025_7500_MOESM1_ESM.pdf]

SUPPLEMENTAL FIGURES AND FIGURE LEGENDS

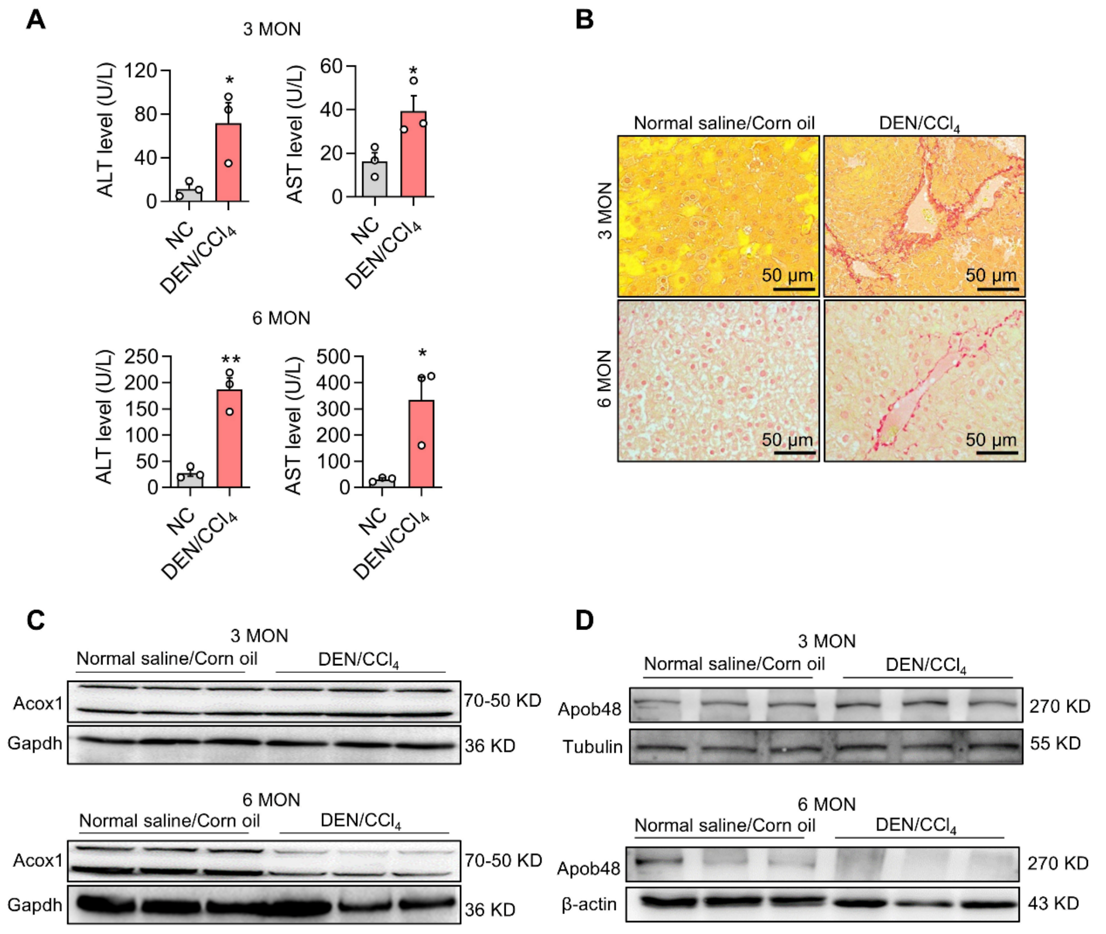

Figure S1. The characterization of DEN/CCl_4-induced HCC models. A, ALT/AST detection for liver function in fibrosis and HCC models. B, Sirius red staining detecting the liver fibrosis at 3 months and 6 months. C, D, Western blot analysis of protein expression involved in fatty acid oxidation (Acox1) and transport (Apob48). *, ** mean $P < 0.05$ and $P < 0.01$.

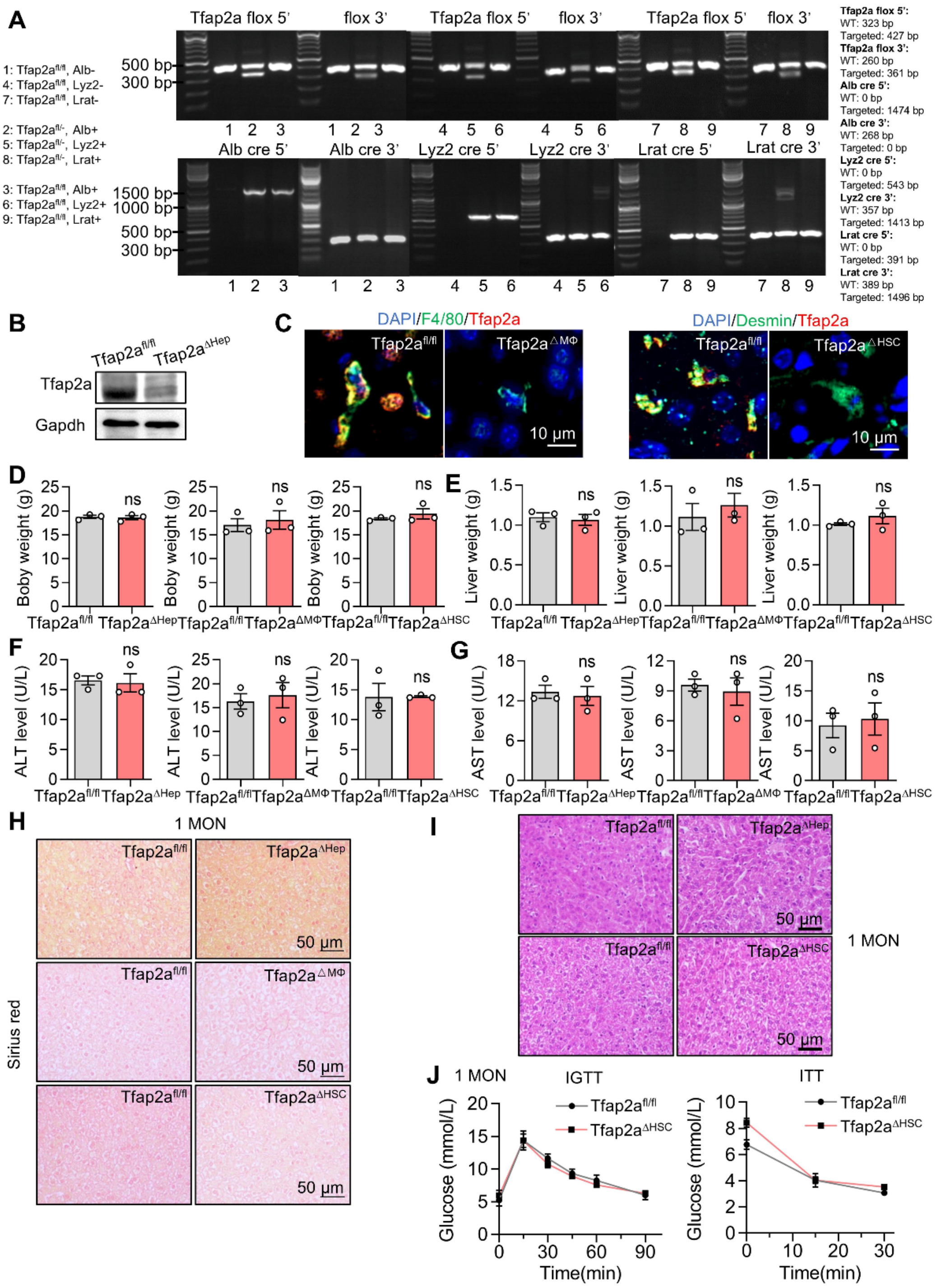

Figure S2. Detection and phenotype analysis of Tfap2a knockout in livers. A, Genotyping of three types of Tfap2a knockout mice. B, C, Western blots and ICC assays verified the knockout efficiency of Tfap2a in three conditional knockout mice. D-G, Effects of Tfap2a knockout in hepatocytes on body weight, liver weight and ALT/AST levels. H, Sirius red staining detecting liver fibrosis in Tfap2a$^{\Delta Hep}$, Tfap2a$^{\Delta M\Phi}$, Tfap2a$^{\Delta HSC}$ mice and corresponding controls after 1 month. I, H&E staining showing

liver morphology and lipid droplet accumulation in the livers of Tfap2a$^{\triangle Hep}$ mice and Tfap2a$^{\triangle HSC}$ mice at 1 month. J, IGTT and ITT analysis of glucose change in Tfap2a$^{\triangle HSC}$ mice and Tfap2a$^{flox/flox}$ mice at 1 month. ns, no significance.

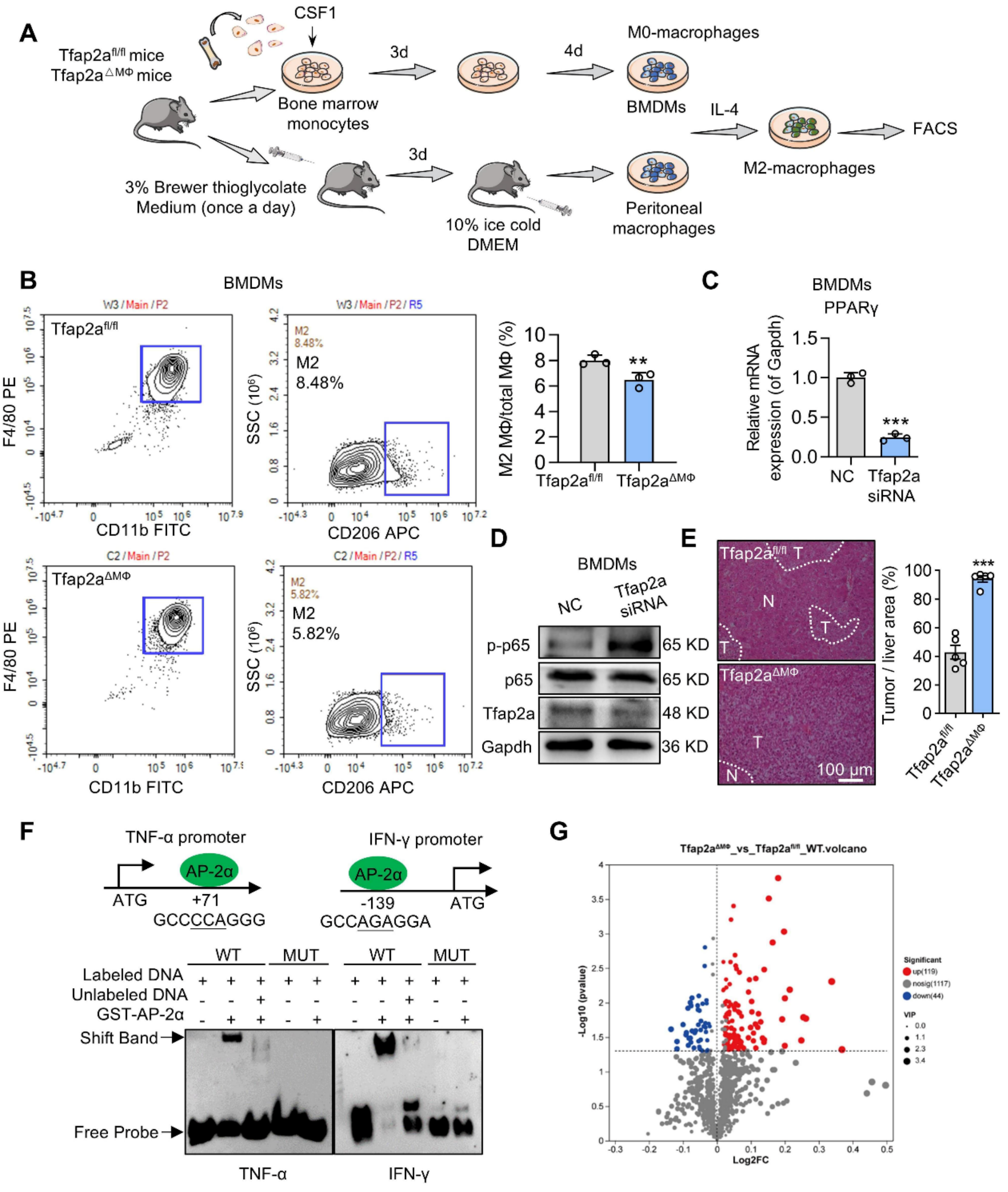

Figure S3. The effects of Tfap2a knockout in macrophages on macrophage polarization. A, The strategy depicting the isolation and treatment of macrophages from Tfap2a$^{\triangle M\Phi}$ mice and Tfap2a$^{fl/fl}$ mice. B, FACS analysis detecting the effects of Tfap2a knockout in BMDMs on the proportion of M2 macrophages. C, D, qRT-PCR

and Western blotting analysis showing the effects of Tfap2a knockout in macrophages on gene expression. E, Analysis of the tumor-to-liver area ratio using H&E staining with ImageJ software. N, Normal. T, Tumor. F, EMSA showing the binding of AP-2α to TNF-α and IFN-γ promoter *in vitro*. G, Non-targeted lipidomics analysis of differential lipids in Tfap2a$^{\triangle M\Phi}$ and Tfap2a$^{fl/fl}$ mouse liver. **, *** mean $P < 0.01$ and $P < 0.001$.

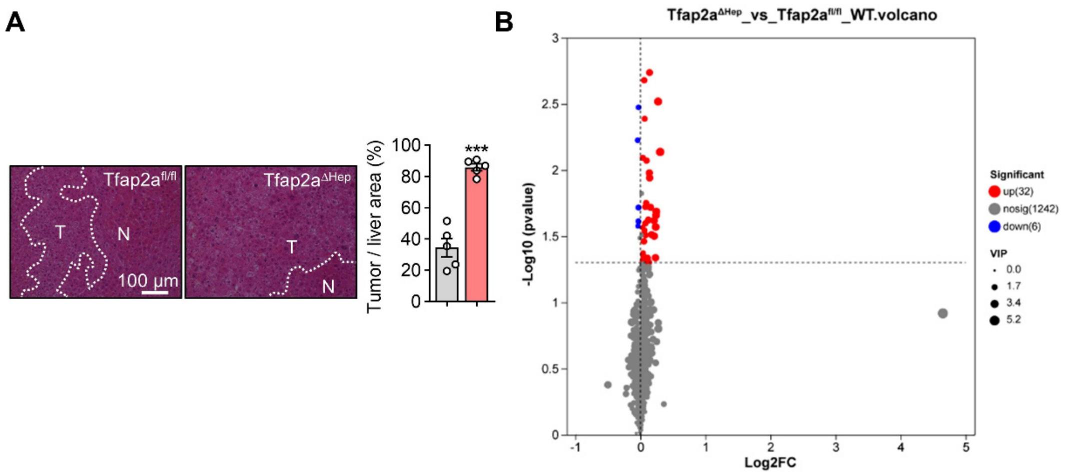

Figure S4. The effects of Tfap2a knockout in hepatocytes on lipid metabolism and tumorigenesis. A, Analysis of the tumor-to-liver area ratio using H&E staining of Tfap2a$^{\triangle Hep}$ and Tfap2a$^{fl/fl}$ mouse liver with ImageJ software. N, Normal. T, Tumor. B, Non-targeted lipidomics analysis of differential lipids in Tfap2a$^{\triangle Hep}$ and Tfap2a$^{fl/fl}$ mouse liver.

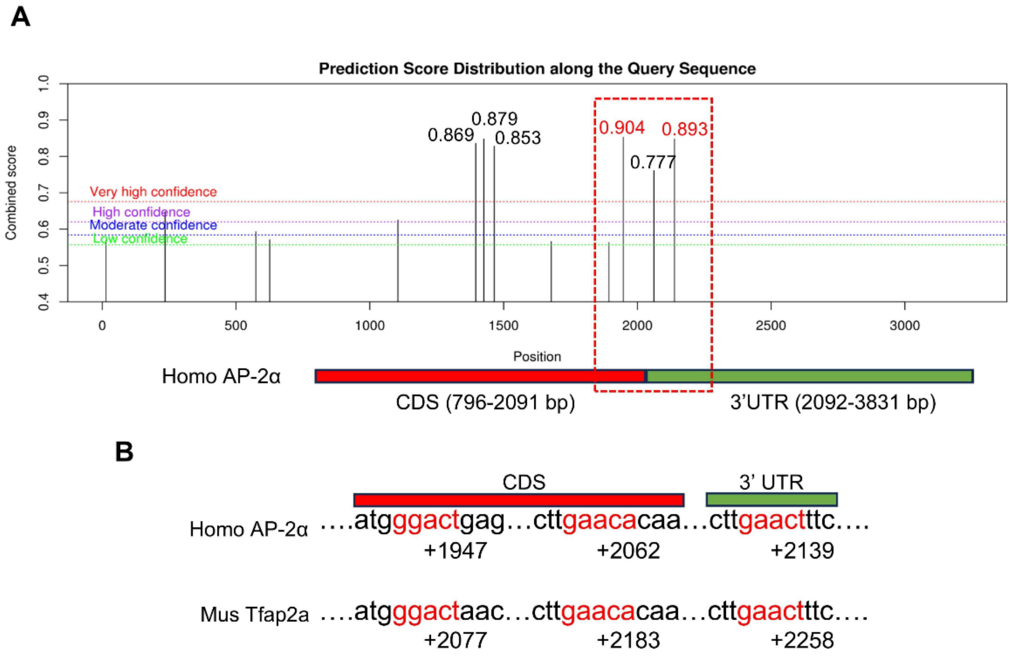

Figure S5. The predicted m6A sites in the AP-2α 3′ UTR. A, The potential m6A sites in the AP-2α 3′ UTR were predicted from the SRAMP website (http://www.cuilab.cn/sramp). B, The conserved m6A sites in the 3′UTR of human AP-2α and mouse Tfap2a.

SUPPLEMENTAL TABLE

Table S1. Primer pairs used in this study.

Name	Sequence(5'-to -3')	Purpose
Acc F	CTCCCGATTCATAATTGGGTCTG	qRT-PCR
Acc R	TCGACCTTGTTTTACTAGGTGC	
Fasn F	CTCTGTTCAGCTATTGGACGC	qRT-PCR
Fasn R	CGGAATTTCTGGGATTCAGCTTC	
Acly F	CAGCCAAGGCAATTTCAGAGC	qRT-PCR
Acly R	CTCGACGTTTGATTAACTGGTCT	
Scd1 F	TCCATCATGAAGTGTGACGT	qRT-PCR
Scd1 R	TACTCCTGCTTGCTGATCCAC	
Acox1 F	GGATGGTAGTCCGGAGAACA	qRT-PCR
Acox1 R	AGTCTGGATCGTTCAGAATCAAG	
Cpt1a F	TGGCATCATCACTGGTGTGTT	qRT-PCR
Cpt1a F	GTCTAGGGTCCGATTGATCTTTG	
Apbo48 F	TTGGCAAACTGCATAGCATCC	qRT-PCR
Apbo48 R	TCAAATTGGGACTCTCCTTTAGC	
Pparγ F	TATGGAGTGACATAGAGTGTGCT	qRT-PCR
Pparγ R	CCACTTCAATCCACCCAGAAAG	
Afp F	CTTCCCTCATCCTCCTGCTAC	qRT-PCR
Afp R	ACAAACTGGGTAAAGGTGATGG	
Gpc3 F	CAGCCCGGACTCAAATGGG	qRT-PCR
Gpc3 R	CAGCCGTGCTGTTAGTTGGTA	
α-Sma F	GTCCCAGACATCAGGGAGTAA	qRT-PCR
α-Sma R	TCGGATACTTCAGCGTCAGGA	
Col1a1 F	GCTCCTCTTAGGGGCCACT	qRT-PCR
Col1a1 R	CCACGTCTCACCATTGGGG	
Col3a1 F	CTGTAACATGGAAACTGGGGAAA	qRT-PCR
Col3a1 R	CCATAGCTGAACTGAAAACCACC	
Col6a1 F	CTGCTGCTACAAGCCTGCT	qRT-PCR
Col6a1 R	CCCCATAAGGTTTCAGCCTCA	
Mmp-2 F	CAAGTTCCCCGGCGATGTC	qRT-PCR

Mmp-2 R	TTCTGGTCAAGGTCACCTGTC	
Tgf-β1 F	TGATACGCCTGAGTGGCTGTCT	qRT-PCR
Tgf-β1 R	CACAAGAGCAGTGAGCGCTGAA	
Il-1β F	TGGACCTTCCAGGATGAGGACA	qRT-PCR
Il-1β R	GTTCATCTCGGAGCCTGTAGTG	
Tnf-α F	GGTGCCTATGTCTCAGCCTCTT	qRT-PCR
Tnf-α R	GCCATAGAACTGATGAGAGGGAG	
Il-10 F	CCCATTCCTCGTCACGATCTC	qRT-PCR
Il-10 R	TCAGACTGGTTTGGGATAGGTTT	
Ifn-γ F	CAGCAACAGCAAGGCGAAAAAGG	qRT-PCR
Ifn-γ R	TTTCCGCTTCCTGAGGCTGGAT	
Tfap2a F	TTTTTCAGCTATGGACCGTCAC	qRT-PCR
Tfap2a R	GAAGTCGGCATTAGGGGTGTG	
Gapdh F	AGGTCGGTGTGAACGGATTTG	qRT-PCR
Gapdh R	TGTAGACCATGTAGTTGAGGTCA	
AP-2α F	AGGTCAATCTCCCTACACGAG	qRT-PCR
AP-2α R	GGAGTAAGGATCTTGCGACTGG	
β-ACTIN F	CACCATTGGCAATGAGCGGTTC	RT-QPCR
β-ACTIN R	AGGTCTTTGCGGATGTCCACGT	
Tfap2a 5’arm F	GGGTGCAAGCATAAAGGGATCAT	Genotyping
Tfap2a 5’arm R	GTTACCAAGCACAGGCATTGCC	
Tfap2a 3’arm F	GCCAGAAATTCAAACCCAGCAA	Genotyping
Tfap2a 3’arm R	TGCGCCAATTAGAGCATCAAAG	
Alb 5’arm F	ATGCCCACCAAAGTCATCAGTGTAG	Genotyping
Alb 5’arm R	TGCTTGTGGAGCAATGTTGGTAG	
Alb 3’arm F	GGGCAGTCTGGTACTTCCAAGCT	Genotyping
Alb 3’arm R	ATATCCCCTTGTTCCCTTTCTGC	
Lyz2 5’arm F	AGTGCTGAAGTCCATAGATCGG	Genotyping
Lyz2 5’arm R	CTGATTCTCCTCATCACCAGG	
Lyz2 3’arm F	AGTGCTGAAGTCCATAGATCGG	Genotyping
Lyz2 3’arm R	GTCACTCACTGCTCCCCTGT	
Lrat 5’arm F	TGAGCCAAGCACTTTGGCTTC	Genotyping
Lrat 5’arm R	TCACATCCTCAGGTTCAGCAGG	

Lrat 3'arm F	TGAGCCAAGCACTTTGGCTTC	Genotyping
Lrat 3'arm R	AACAGGATGTCAGGCATTAGATGG	
ACC-Promoter F	GGTACCCGGGGTACCACGTCCAGTAAAGGATGCACA	PCR
ACC-Promoter R	AAGCTTCCCAAGCTTTTGGGACATACCTAGCCCTCA	
FASN-Promoter F	GGTACCGCATTCCCATCCCGCACACGT	PCR
FASN-Promoter R	AAGCTTGAAGCGAAGGCGGCTGTT	
SREBP-Promoter F	GGTACCATCAGGCCTGCTCACAACAC	PCR
SREBP-Promoter R	AAGCTTTGGCATTCGGTGGATGTCAG	
IL-10-Promoter F	CGGGGTACCCTAGGAACACGCGAATGAGA	PCR
IL-10-Promoter R	CCCAAGCTTGGAGAATGTCTAGTTCAGGC	
FASN (+293) WT F	CGGAGGCGCCCGGGGCCCCGCGG	EMSA Probe
FASN (+293) WT R	CCGCGGGGCCCCGGGCGCCTCCG	
FASN (+293) MUT F	CGGAGGCAATCGGGTACCCGCGG	EMSA Probe
FASN (+293) MUT R	CCGCGGGTACCCGATTGCCTCCG	
FASN (-283) WT F	CGTCGGGGCCCGCGGGGCGGGAG	EMSA Probe
FASN (-283) WT R	CTCCCGCCCCGCGGGCCCCGACG	
FASN (-283) MUT F	CGTCGGGAATCGCGTAGCGGGAG	EMSA Probe
FASN (-283)	CTCCCGCTACGCGATTCCCGACG	

MUT R		
ACC(-953) WT F	GGCCCACGCCTCAGGCACGAGAATC	EMSA Probe
ACC(-953) WT R	GATTCTCGTGCCTGAGGCGTGGGCC	
ACC (-953) MUT F	GGCCCACGATTCAGTAACGAGAATC	EMSA Probe
ACC(-953) MUT R	GATTCTCGTTACTGAATCGTGGGCC	
ACC (-401) WT F	CGGGGCGGGCCCCCGGGGTCAGGC	EMSA Probe
ACC (-401) WT R	GCCTGACCCCGGGGGCCCGCCCCG	
ACC (-401) MUT F	CGGGGCGGGATCCCGTAGTCAGGC	EMSA Probe
ACC (-401) MUT R	GCCTGACTACGGGATCCCGCCCCG	
ACC (-101) WT F	GCGTGCGCGCCCCCGGGCGGCGC	EMSA Probe
ACC (-101) WT R	GCGCCGCCCGGGGGCGCGCACGC	
ACC (-101) MUT F	GCGTGCGCGATCCCGTACGGCGC	EMSA Probe
ACC (-101) MUT R	GCGCCGTACGGGATCGCGCACGC	
ACC (+140) WT F	GGCCCGTTGCCTGAGGCTTCCTGGC	EMSA Probe
ACC (+140) WT R	GCCAGGAAGCCTCAGGCAACGGGCC	
ACC (+140) MUT F	GGCCCGTTGATTGAGTATTCCTGGC	EMSA Probe
ACC (+140) MUT R	GCCAGGAATACTCAATCAACGGGCC	

SREBP1(-485) WT F	TGGGCCCAGGCCTGGGGCTTCCACT	EMSA Probe
SREBP1(-485) WT R	AGTGGAAGCCCCAGGCCTGGGCCCA	
SREBP1(-485) MUT F	TGGGCCCAGTACTGGATCTTCCACT	EMSA Probe
SREBP1(-485) MUT R	AGTGGAAGATCCAGTACTGGGCCCA	
SREBP1(+496) WT F	CGGTCATTCCCTCCGGCCCGAGA	EMSA Probe
SREBP1(+496) WT R	TCTCGGGCCGGAGGGAATGACCG	
SREBP1(+496) MUT F	CGGTCATTTACTCCGATCCGAGA	EMSA Probe
SREBP1(+496) MUT R	TCTCGGATCGGAGTAAATGACCG	
IL-10(-1338) WT F	CAGCCCAGGCCAGGGCACCCA	EMSA Probe
IL-10(-1338) WT R	TGGGTGCCCTGGCCTGGGCTG	
IL-10(-1338) MUT F	CAGCCCAGATCAGTACACCCA	EMSA Probe
IL-10(-1338) MUT R	TGGGTGTACTGATCTGGGCTG	
TNF-α(+71) WT F	CAGGGGGGCCCCAGGGCTCCAG	EMSA Probe
TNF-α(+71) WT R	CTGGAGCCCTGGGGCCCCCCTG	
TNF-α(+71) MUT F	CAGGGGGTAACCATTTCTCCAG	EMSA Probe
TNF-α(+71) MUT R	CTGGAGAAATGGTTACCCCCTG	
IFN-γ(-139) WT	ACCAGCAGCCAGAGGAGGTGC	EMSA

F		Probe
IFN-γ(-139) WT R	GCACCTCCTCTGGCTGCTGGT	
IFN-γ(-139) MUT F	ACCAGCAGATAGATAAGGTGC	EMSA Probe
IFN-γ(-139) MUT R	GCACCTTATCTATCTGCTGGT	
FASN 131 F	TGGCCCAAGCATTCCCATC	ChIP
FASN 131 R	GACTTCCGCCTCCCGC	
FASN 165 F	TCCTCATCCTCCGCTCTCG	ChIP
FASN 165 R	TGTTGGTGGCTTTCCCCG	
ACC 182 F	CCCCTTTCACAAGAGACCACA	ChIP
ACC 182 R	GAACGTTGGCTCCACAACTC	
SREBP1+496 F	CCGTGACGTAATTGCGAGGT	ChIP
SREBP1+496 R	TGGCATTCGGTGGATGTCAG	
SREBP1-485 F	GGCCTGGGACCCCTATAACT	ChIP
SREBP1-485 R	TGTTTCTCCAGCACCAAGCA	
METTL3	CUGCAAGUAUGUUCACUAUGA	siRNA
	UCAUAGUGAACAUACUUGCAG	
ALKBH5	ACAAGUACUUCUUCGGCGA(dT)(dT)	siRNA
	UCGCCGAAGAAGUACUUGU(dT)(dT)	
WTAP	AAGCUUUGGAGGGCAAGUACA(dT)(dT)	siRNA
	UGUACUUGCCCUCCAAAGCUU(dT)(dT)	
METTL14	AAGGAUGAGUUAAUAGCUAAA	siRNA
	UUUAGCUAUUAACUCAUCCUU	
FTO	AUAGCCGCUGCUUGUGAGATT	siRNA
	UCUCACAAGCAGCGGCUAUUU	
YTHDF1	CCGCGUCUAGUUGUUCAUGAA	siRNA
	UUCAUGAACAACUAGACGCGG	
YTHDF2	AAGGACGUUCCCAAUAGCCAA	siRNA
	UUGGCUAUUGGGAACGUCCUU	
YTHDF3	UAAGUCAAAGAAGACGUAUUA	siRNA
	UAAUACGUCUUCUUUGACUUA	

YTHDC1	CGACCAGAAGAUUAUGAUAUU	siRNA
	AAUAUCAUAAUCUUCUGGUCG	
YTHDC2	GCCCACAGAUUGGCUUAUUUA	siRNA
	UAAAUAAGCCAAUCUGUGGGC	
Mus Tfap-2a	GCUCCACCUCGAAGUACAA(dT)(dT)	siRNA
	UUGUACUUCGAGGUGGAGC(dT)(dT)	
Homo TFAP-2α	GCAAGAUCCUUACUCCCACTT	siRNA
	GUGGGAGUAAGGAUCUUGCTT	
TFAP2a 3'UTR F	GGACTGAGTCACCACCTTCC	MeRIP/RIP
TFAP2a 3'UTR R	CTGCGAATCGTGTTGCCAG	MeRIP/RIP

Table S2. AP-2α expression and clinical characteristics

Clinical features	Number	AP-2a			P Value
		Overexpression	Low expression	No expression	
Total number	60	16	14	30	
Gender					0.9671
Female	20	5	5	10	
Male	40	11	9	20	
Age (median,51.5 years)					0.157
<	30	6	10	14	
⩾	30	9	5	16	
Histological diagnosis					**<0.000**1
Hepatitis	13	4	6	3	
Non-alcoholic fatty liver	10	5	2	3	
Cirrhosis	12	2	2	8	
Hepatocellular carcinoma	20	0	5	15	
HCC histological Grade					**<0.000**1
Grade I	3	0	1	2	
Grade II	10	0	4	6	
Grade III	7	0	0	7	
Normal liver tissue	5	5	0	0	

Table S3. WTAP expression and clinical characteristics

Clinical features	Number	WTAP			P Value
		Overexpression	Low expression	No expression	
Total number	60	25	27	8	
Gender					0.517
Female	20	7	9	4	
Male	40	18	18	4	
Age (median,51.5 years)					0.400
<	30	10	15	5	
⩾	30	15	12	3	
Histological diagnosis					0.102
Hepatitis	13	5	6	2	
Non-alcoholic fatty liver	10	6	4	0	
Cirrhosis	12	6	5	1	
Hepatocellular carcinoma	20	8	10	2	
HCC histological Grade					**0.008**
Grade I	3	3	0		
Grade II	10	1	7	2	
Grade III	7	4	3		
Normal liver tissue	5	0	2	3	

Table S4. YTHDC1 expression and clinical characteristics

Clinical features	Number	YTHDC1			P Value
		Overexpression	Low expression	No expression	
Total number	60	36	21	3	
Gender					>0.999
Female	20	12	7	1	
Male	40	24	14	2	
Age (median,51.5 years)					0.283
<	30	21	9	0	
⩾	30	15	13	2	
Histological diagnosis					**0.037**
Hepatitis	13	6	7	0	
Non-alcoholic fatty liver	10	7	3	0	
Cirrhosis	12	6	6	0	
Hepatocellular carcinoma	20	17	3	0	
HCC histological Grade					**0.004**
Grade I	3	2	1	0	
Grade II	10	8	2	0	
Grade III	7	7	0	0	
Normal liver tissue	5	0	2	3	

Table S5. FASN expression and clinical characteristics

Clinical features	Number	FASN Overexpression	FASN Low expression	FASN No expression	P Value
Total number	60	40	18	2	
Gender					0.675
Female	20	14	5	1	
Male	40	25	14	1	
Age (median,51.5 years)					0.111
<	30	22	6	2	
⩾	30	18	12	0	
Histological diagnosis					0.068
Hepatitis	13	8	5	0	
Non-alcoholic fatty liver	10	8	2	0	
Cirrhosis	12	8	4	0	
Hepatocellular carcinoma	20	16	4	0	
HCC histological Grade					**0.007**
Grade I	3	3	0	0	
Grade II	10	9	1	0	
Grade III	7	6	1	0	
Normal liver tissue	5	0	3	2	
