# Supplementary material for: Deletion of Tfap2a in hepatocytes and macrophages promotes the progression of hepatocellular carcinoma by regulating SREBP1/FASN/ACC pathway and anti-inflammatory effect of IL10
Source: Cell Death Dis. 2025 Apr 3;16(1):245. doi: 10.1038/s41419-025-07500-8 (PMC11968862; doi:10.1038/s41419-025-07500-8)

Fig.1H

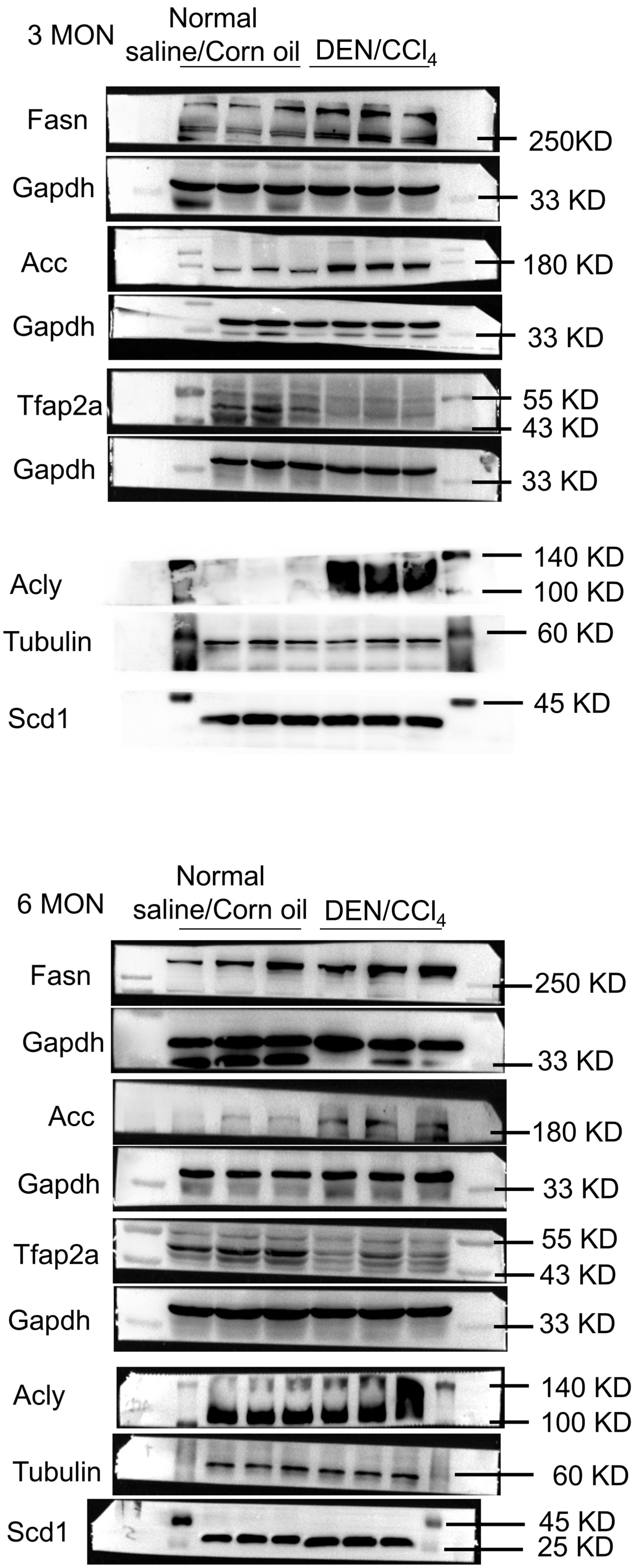
3 MON
Normal saline/Corn oil
DEN/CCl4
Fasn
Gapdh
Acc
Gapdh
Tfap2a
Gapdh
Acly
Tubulin
Scd1
250KD
33 KD
180 KD
33 KD
55 KD
43 KD
33 KD
140 KD
100 KD
60 KD
45 KD
6 MON
Normal saline/Corn oil
DEN/CCl4
Fasn
Gapdh
Acc
Gapdh
Tfap2a
Gapdh
Acly
Tubulin
Scd1
250 KD
33 KD
180 KD
33 KD
55 KD
43 KD
33 KD
140 KD
100 KD
60 KD
45 KD
25 KD

Fig.S1C

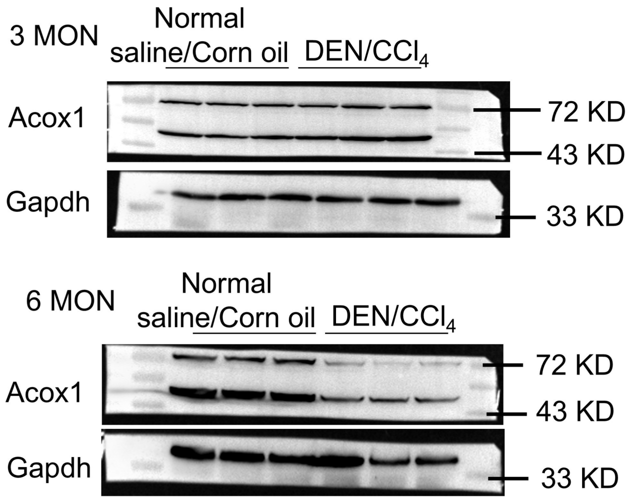

Fig.S1D

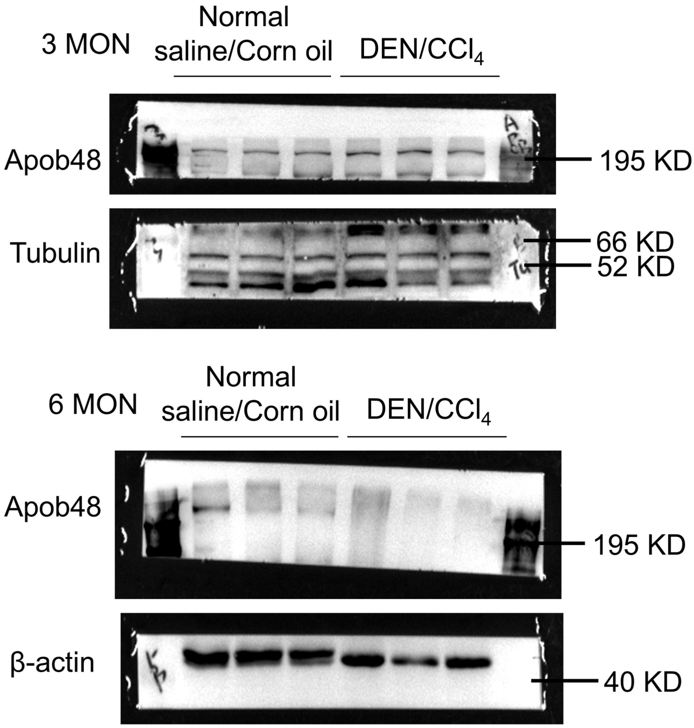

Fig.S2A

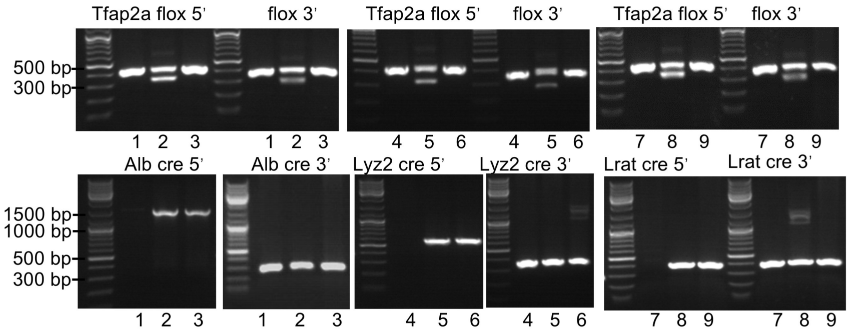

Fig.S2B

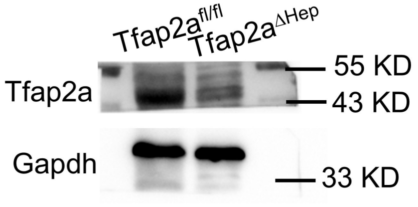

Fig.3F

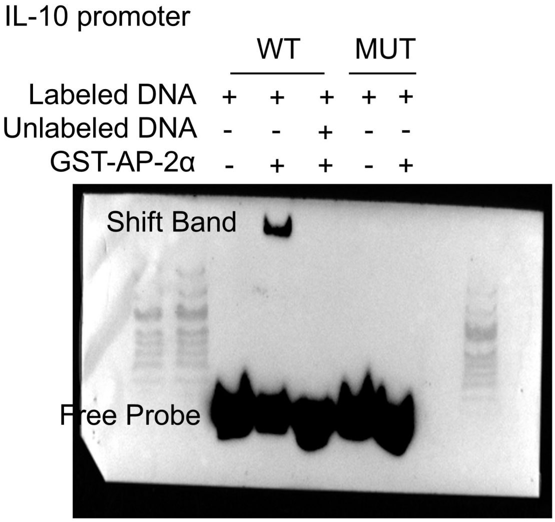

Fig.3O

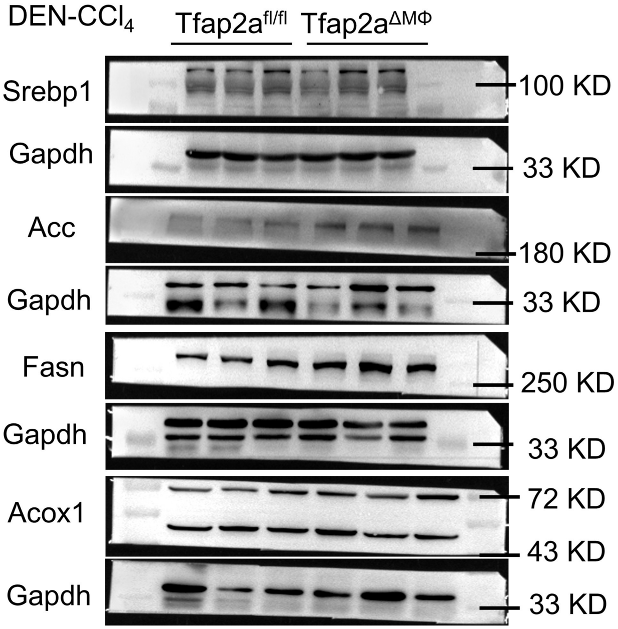

Fig.S3D

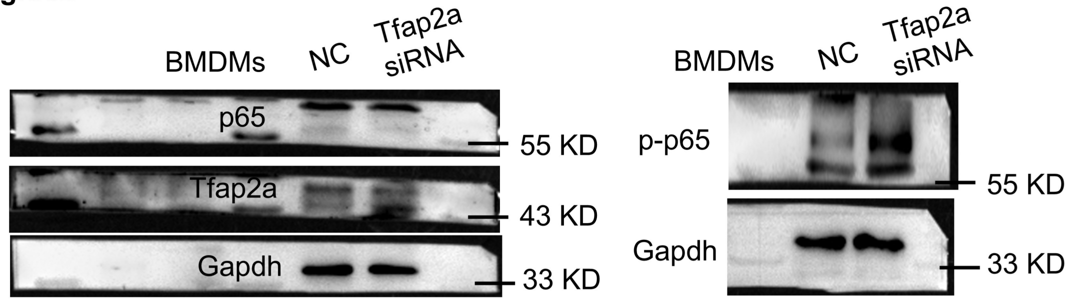

Fig.S3F

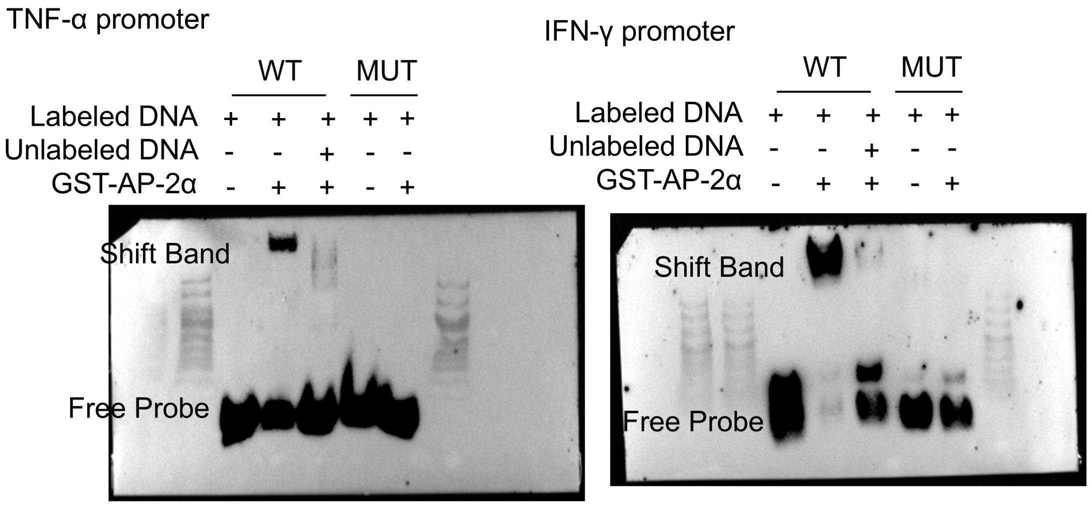

Fig.4J

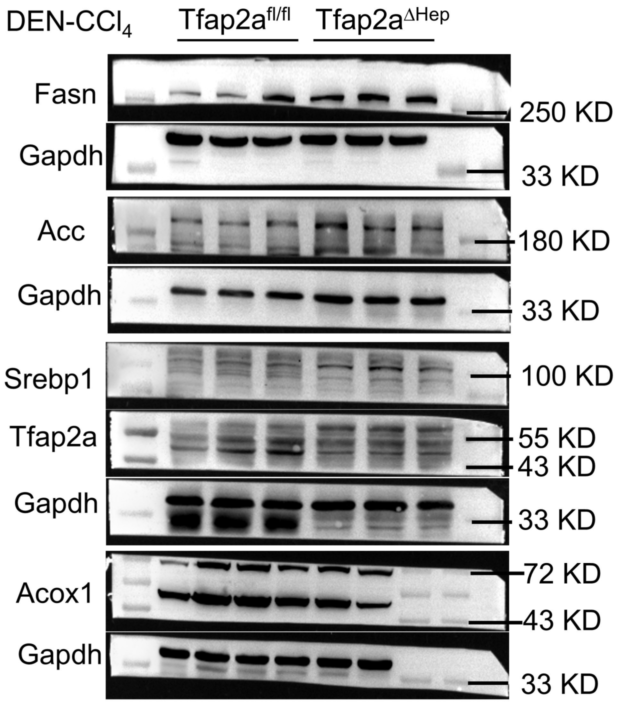

DEN-CCl4
Tfap2afl/fl
Tfap2aΔHep
Fasn
250 KD
Gapdh
33 KD
Acc
180 KD
Gapdh
33 KD
Srebp1
100 KD
Tfap2a
55 KD
43 KD
Gapdh
33 KD
Acox1
72 KD
43 KD
Gapdh
33 KD

Fig.5C

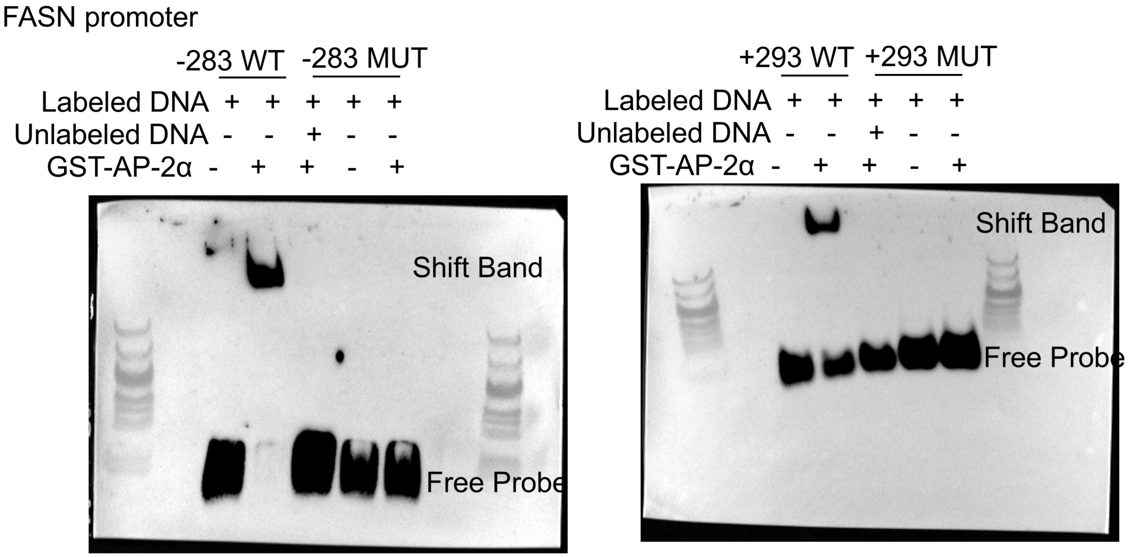

Fig.5D

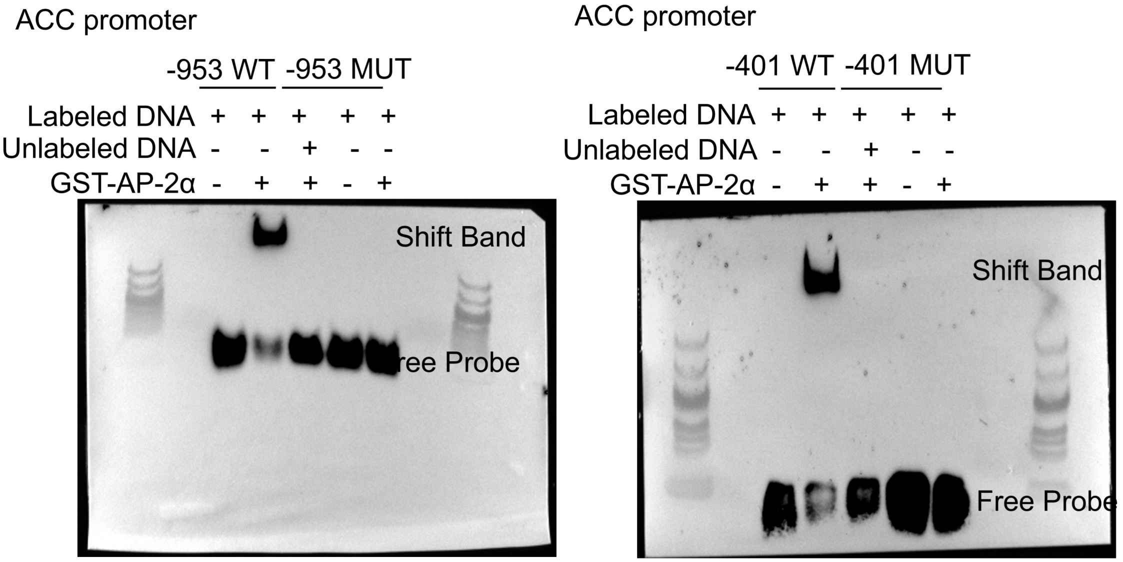

Fig.5E

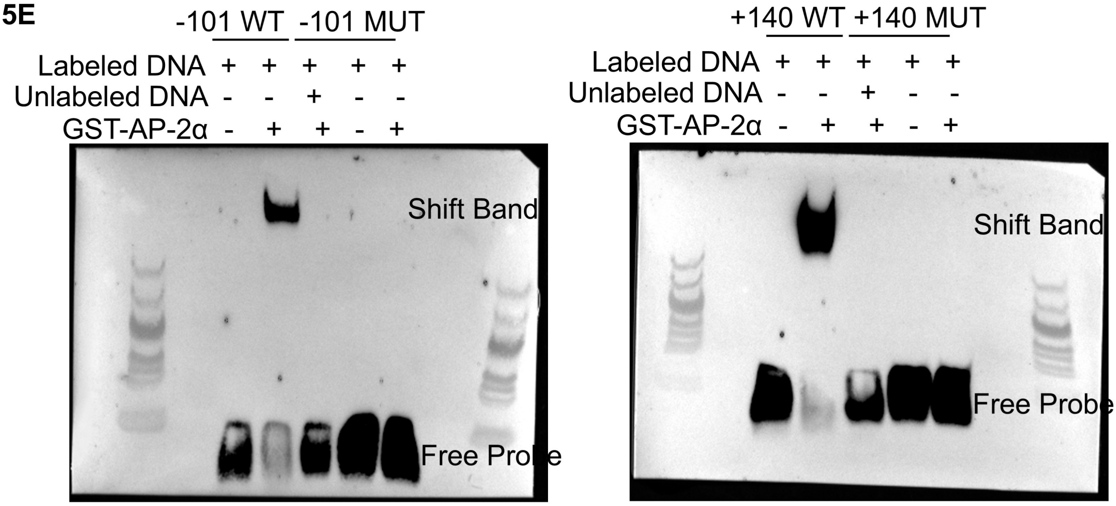

Fig.5F

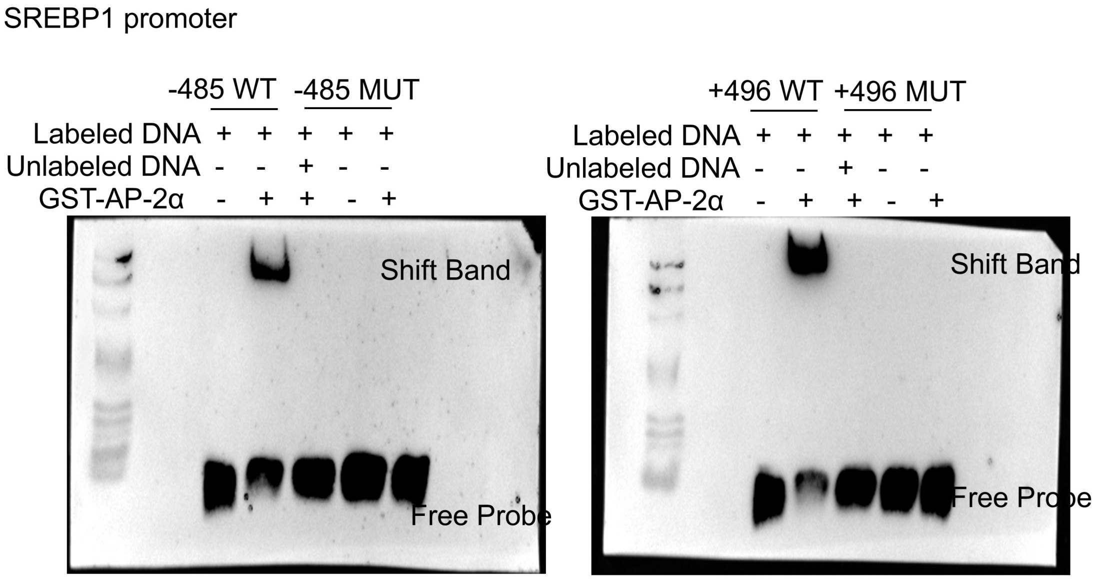

Fig. 5G

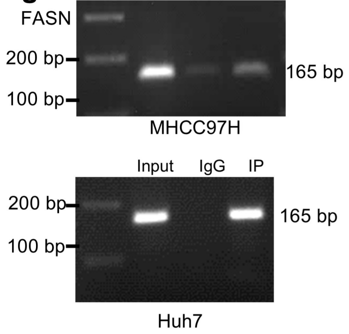

Fig. 5H

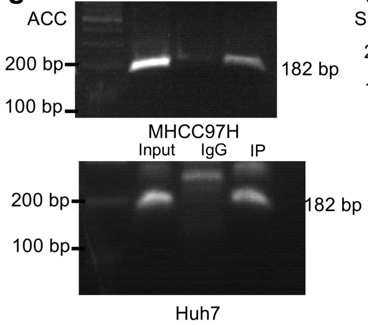

Fig. 5I

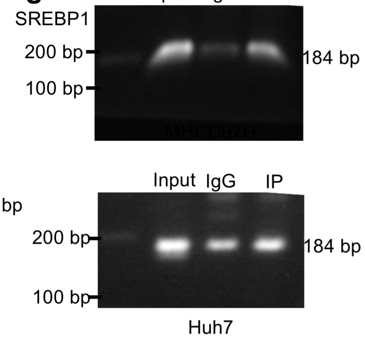

Fig.5J

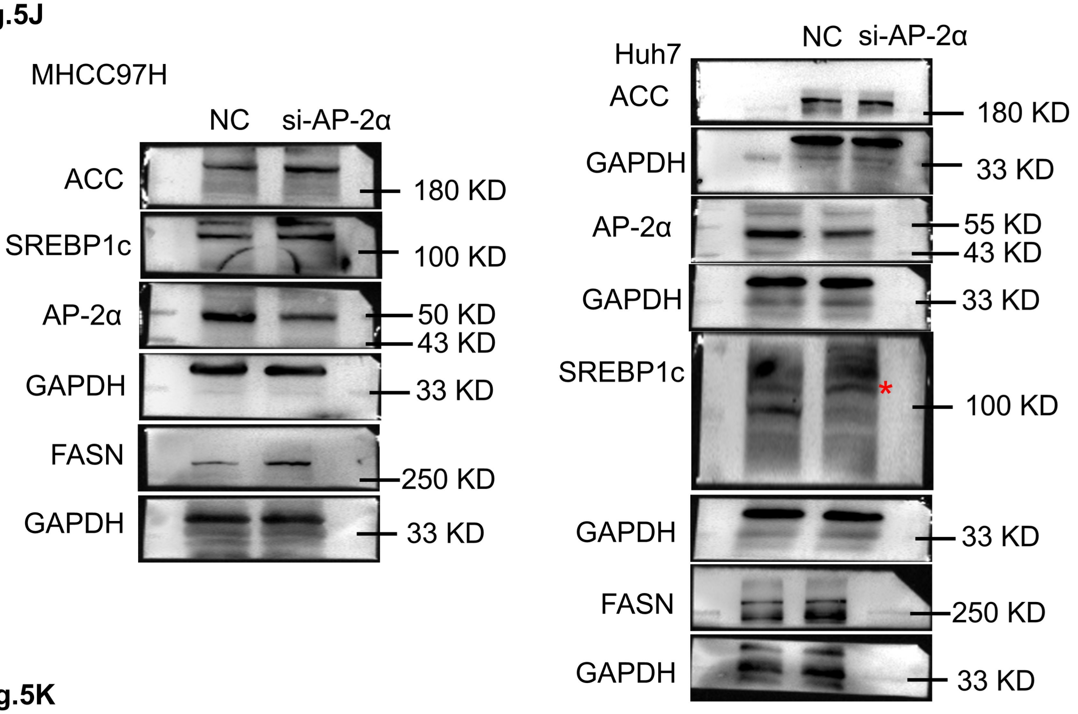
MHCC97H
NC
si-AP-2α
ACC
180 KD
SREBP1c
100 KD
AP-2α
50 KD
43 KD
GAPDH
33 KD
FASN
250 KD
GAPDH
33 KD
Huh7
NC
si-AP-2α
ACC
180 KD
GAPDH
33 KD
AP-2α
55 KD
43 KD
GAPDH
33 KD
SREBP1c
*
100 KD
GAPDH
33 KD
FASN
250 KD
GAPDH
33 KD

Fig.5K

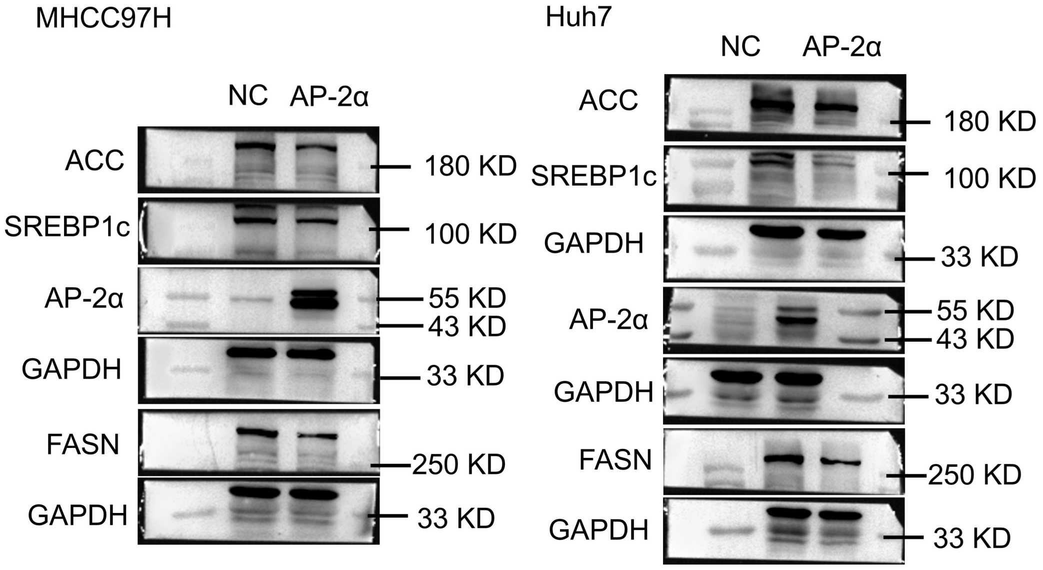
MHCC97H
NC
AP-2α
ACC
180 KD
SREBP1c
100 KD
AP-2α
55 KD
43 KD
GAPDH
33 KD
FASN
250 KD
GAPDH
33 KD
Huh7
NC
AP-2α
ACC
180 KD
SREBP1c
100 KD
GAPDH
33 KD
AP-2α
55 KD
43 KD
GAPDH
33 KD
FASN
250 KD
GAPDH
33 KD

Fig.6A

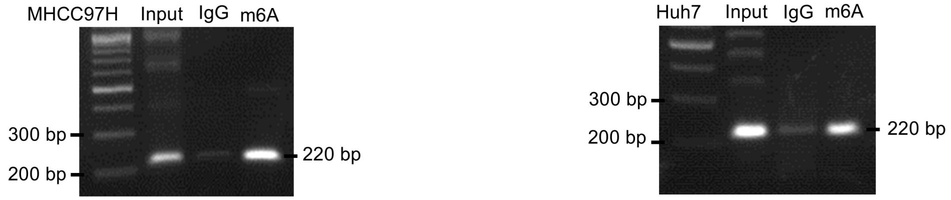

Fig.6B

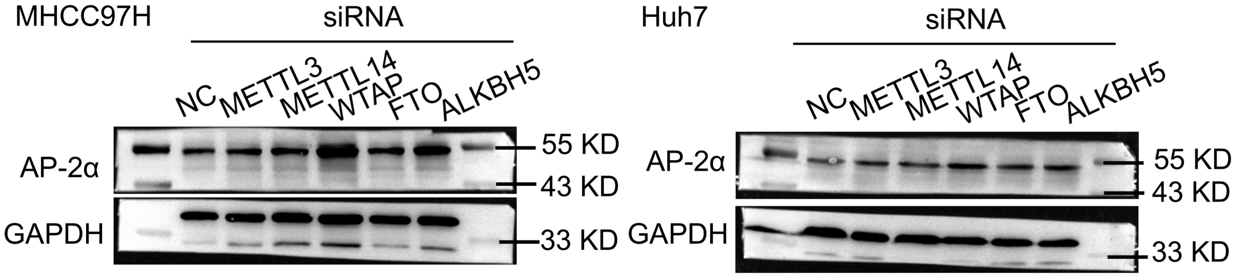

Fig. 6E

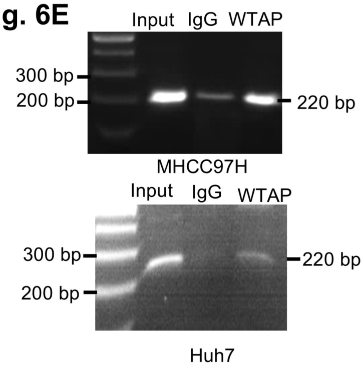

Fig. 6I

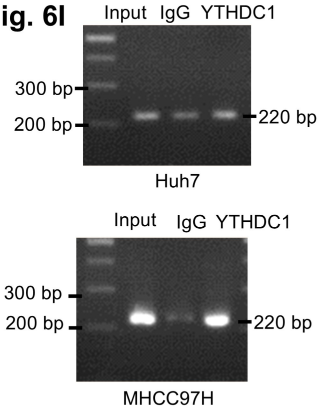

Fig.6G

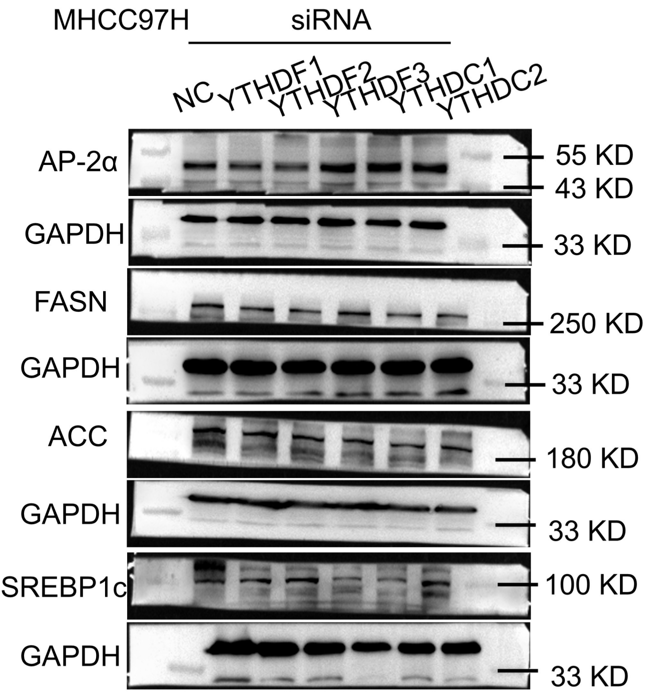

Fig.6L

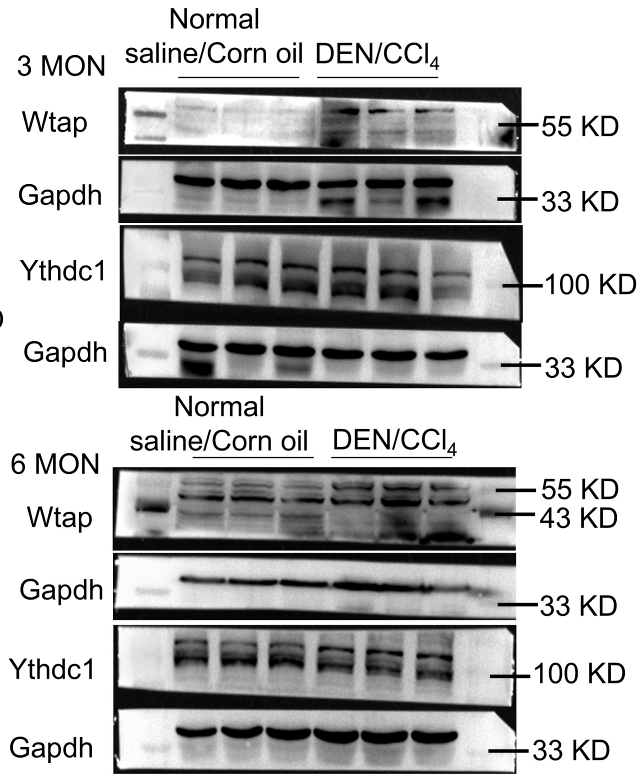

Supplement: Supplementary file 2 — Original data of WB [file 41419_2025_7500_MOESM2_ESM.pdf]
